# Distinct and overlapping gene regulatory networks in BMP- and HDAC-controlled cell fate determination in the embryonic forebrain

**DOI:** 10.1186/1471-2164-13-298

**Published:** 2012-07-02

**Authors:** Catharina Scholl, Kathrin Weiβmüller, Pavlo Holenya, Maya Shaked-Rabi, Kerry L Tucker, Stefan Wölfl

**Affiliations:** 1 Institute of Pharmacy and Molecular Biotechnology, Heidelberg University, 69120, Heidelberg, Germany; 2 Interdisciplinary Center for Neurosciences, Heidelberg University, 69120, Heidelberg, Germany; 3 Institute of Anatomy and Cell Biology, University of Heidelberg, 69120, Heidelberg, Germany; 4 The National Hospital for Neurology & Neurosurgery, Institute of Neurology, UCL, London, WC1 N3BG, UK

## Abstract

**Background:**

Both bone morphogenetic proteins (BMPs) and histone deacetylases (HDACs) have previously been established to play a role in the development of the three major cell types of the central nervous system: neurons, astrocytes, and oligodendrocytes. We have previously established a connection between these two protein families, showing that HDACs suppress BMP-promoted astrogliogenesis in the embryonic striatum. Since HDACs act in the nucleus to effect changes in transcription, an unbiased analysis of their transcriptional targets could shed light on their downstream effects on BMP-signaling.

**Results:**

Using neurospheres from the embryonic striatum as an *in vitro* system to analyze this phenomenon, we have performed microarray expression profiling on BMP2- and TSA-treated cultures, followed by validation of the findings with quantitative RT-PCR and protein analysis. In BMP-treated cultures we first observed an upregulation of genes involved in cell-cell communication and developmental processes such as members of BMP and canonical Wnt signaling pathways. In contrast, in TSA-treated cultures we first observed an upregulation of genes involved in chromatin modification and transcription. Interestingly, we could not record direct changes in the protein levels of canonical members of BMP2 signaling, but we did observe an upregulation of both the transcription factor STAT3 and its active isoform phospho-STAT3 at the protein level.

**Conclusions:**

STAT3 and SMAD1/5/8 interact synergistically to promote astrogliogenesis, and thus we show for the first time that HDACs act to suppress BMP-promoted astrogliogenesis by suppression of the crucial partner STAT3.

## Background

During development of the central nervous system a variety of different cell-types need to be generated. The three major brain cell types, neurons, astrocytes and oligodendrocytes, arise from neural progenitor cells. Neurons are the first cell type to be generated, starting soon after formation of the neuroectoderm at mid-gestation, and astrocytes and oligodendrocytes are born only shortly before birth and continuing into the postnatal period. The mechanisms by which neural stem cells transition from a neuron- to an astrocyte-generating progenitor are only partially understood, but secreted growth factors are known to play a role in this process. For example, multiple bone morphogenetic proteins (BMPs), members of the TGF-beta super family, and their receptors are abundantly expressed in the developing brain, starting as early as 8.75 days *post coitum* (E8.75) [1-4]. *In vitro*, BMPs were shown to promote the generation of astrocytes [5], and *in vivo*, shown to promote astrocyte formation at the expense of oligodendrocytes [6,7]. In particular, BMP2/4 are known to enhance astrogliogenesis and to inhibit neurogenesis through induction of the inhibitory basic helix-loop-helix transcription factor genes *Id1Id3*, and *Hes5* which antagonize the proneural gene *Ngn1*[8]. However, BMP2/4 has also been shown to promote neuronal differentiation in the cortex [9,10].

It is becoming increasingly evident that the regulation of genes involved in brain development occurs not just at the level of the expression of activating and inhibiting transcription factors, but also at the epigenetic level, in the covalent modification of chromatin [11]. Core histones can be methylated, phosphorylated, ubiquitinated and acetylated, to name just the best-known chemical groups involved, and these small moieties regulate the chromatin structure and subsequent gene expression. Acetylation of the ε-amino groups of lysine residues in the amino-termini of core histones by histone acetyltransferases (HATs) leads to relaxation of chromatin conformation, resulting in transcriptional activation [12]. Conversely, histone deacetylation increases chromatin compaction and thereby reduces accessibility of transcription factors to the DNA. Deacetylation is catalyzed by histone deacetylases (HDAC), a large group of enzymes which are classified, based upon their domain structure and sequence homology, into four gene families [13]. Class I HDACs (HDAC1, -2, -3, and −8) are orthologs of the yeast transcriptional regulator RPD3 and are primarily localized in the nucleus. Class II HDACs (HDAC4, -5, -6, -7, -9, and −10) are homologous to the yeast HDA1 protein and can shuttle between the nucleus and the cytoplasm. Structurally and mechanistically different classes of HDACs are the sirtuins (Sirt1-7), also known as Class III HDACs. They are NAP-depended enzymes homologous to yeast Sir2 (silent information regulator 2) [14]. HDAC11 is the only histone deacetylase categorized to HDAC class IV [15].

It has been previously shown that histone acetylation is crucial for the dynamic regulation of gene expression during differentiation processes. Especially, skeletal and cardiac myogenesis have been intensively studied [16]. Recent publications strongly suggest that HDACs are also important for the development of the nervous system. A large number of different HDACs are expressed in the developing brain, suggesting specific roles for individual HDACs in neural development [17]. HDACs have been shown to be involved in the birth and maturation of oligodendrocytes in the rat, mouse, and in zebrafish [18-21]. It has also been shown that HDACs play an important role in the control of neurogenesis and astrogliogenesis. Especially HDAC1 and HDAC2 have been reported in the regulation of distinct linage specification in developing brain. During neuronal development HDAC1 and 2 are both expressed in stem and progenitor cells. In post-mitotic neurons only HDAC2 expression can be detected, while HDAC1 is only expressed in glia [22]. Deletion of both HDAC1 and 2 results in major abnormalities in cortical, hippocampal and cerebellar development, whereas an individual deletion of HDAC1 or HDAC2 has no effect. Interestingly, deletion of HDAC1 and HDAC2 almost completely blocks the neuronal differentiation, but does not influence astrogliogenesis [23].

Trichostatin A (TSA), a well-established reversible inhibitor of class I and II HDACs [24], has been reported to induce cell growth arrest, apoptosis and differentiation in tumor cells [25]. The treatment of adult neural progenitor cells with HDAC inhibitors causes antiproliferative effects and induces neuronal differentiation, whereas the differentiation of astrocytes or oligodendrocytes is simultaneously not induced [26]. In a previous study we could demonstrate that inhibition of class I and II HDACs with TSA leads to an increase in neurogenesis in the developing cortex, but results in a dramatic reduction in neurogenesis in the medial and lateral ganglionic eminences (GE) of the embryonic forebrain [27]. The reduction in neurogenesis in GE-derived neural precursors was accompanied by an increase in the production of immature astrocytes. We could further demonstrate that treatment with recombinant BMP2 increased the production of astrocytes in neural precursors derived from GE, whereas no significant increase in astrogliogenesis was detected in cortical neural precursor cells. A co-treatment with TSA and noggin, a BMP2 inhibitor, or with Alk3-ECD, a recombinant protein that contains the extracellular domain of the BMPR1A receptor, was able to restore the normal levels of neurons and astrocytes, compared to untreated control samples, demonstrating a direct connection between HDAC activity and BMP signaling [27]. In order to investigate the signaling pathways involved in the differentiation of GE derived neural precursors upon TSA and BMP2 treatment, we performed gene expression profiling and protein analysis from BMP2 or TSA treated neural precursor cells derived from GE at different time points. Here, we show that BMP2 and TSA influence neurogenesis in a related manner. We demonstrate that in the early response to BMP2 and TSA treatment, different cohorts of functional gene groups are activated or repressed, although the downstream biological effects are closely related. We further characterized individual genes picked up by the microarrays at both mRNA and protein levels.

## Results

### *In vitro* differentiation of forebrain derived neurosphere cultures

**Figure 1:**
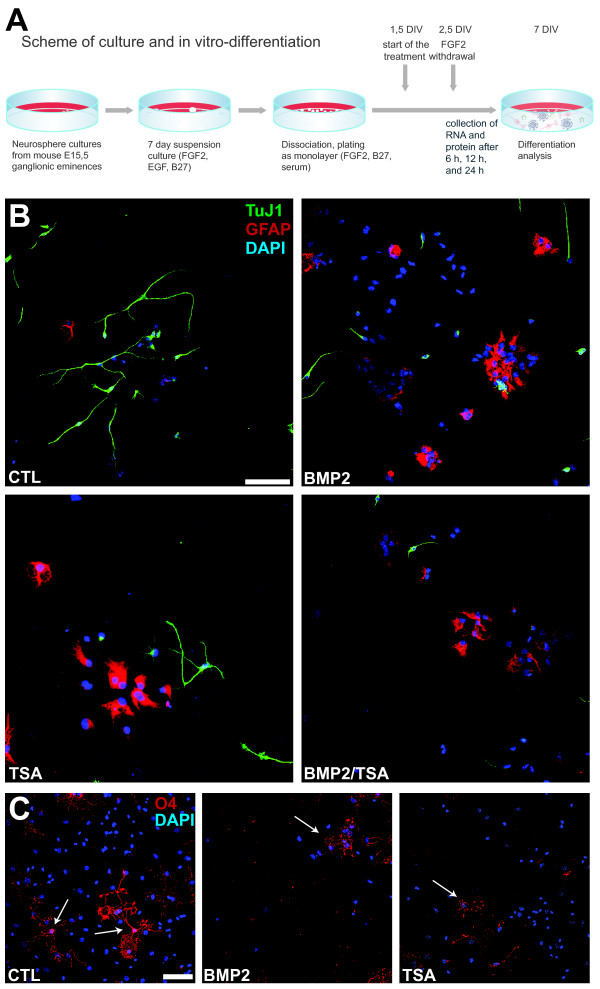
** Neurosphere cultures and immunocytofluorescence.** For *in vitro* differentiation cells from the basal ganglia of 15.5 dpc C57BL/6 mice were cultured in neurospheres and dissociated after 7 days. FGF2 was withdrawn after 2.5 days and treatment started 1.5 days after plating. Cells were treated with TSA (10, 25 or 50nM) or BMP2 (10 ng/ml). RNA and proteins were isolated after 6, 12 and 24 h (**A**). For immunocytofluorescence (**B**,**C**), cultures were treated with vehicle (CTL), 50nM trichostatin A (TSA), 10 ng/ml recombinant BMP2 (BMP2), or both reagents (BMP2/TSA) for 24 hours before bFGF withdrawal. Cultures were fixed after 4.5 additional days and stained with the following antibodies: TuJ1 (B, green) to label newborn neurons, anti-GFAP (B, red) to label newborn astrocytes, or O4 (C, red, indicated with arrows) to label newborn oligodendrocytes. DAPI (blue) was used to stain nuclei. Scale bar = 50 (**B**) and 100 (**C**) μm.

We used neurosphere cultures to generate a uniform population of neural precursors directly from the medial and lateral ganglionic eminences of E15.5 C57BL/6 mice [28]. After 7 days neurospheres were dissociated, plated out as a monolayer, and differentiated according to standard protocols [29]. During differentiation FGF2 was withdrawn after 2.5 days, whereas the treatment with TSA or BMP2 started 1.5 days after plating (Figure 1A).

Cultures were allowed to differentiate for an additional 4.5 days after FGF2 withdrawal and then stained with immunocytofluorescence for standard markers indicating the birth of newborn neurons (TuJ1), astrocytes (GFAP), and oligodendrocytes (O4) (Figure 1B,C). As reported previously [27], both TSA as well as BMP2 treatment suppressed neurogenesis and boosted astrogliogenesis, as indicated by the relative number of TuJ1-positive neurons and GFAP-positive astrocytes in the cultures (Figure 1B). Simultaneous treatment with both TSA and BMP2 showed a similar effect (Figure 1B). As reported previously [27], both TSA as well as BMP2 treatment suppressed the birth and maturation of oligodendrocytes, as judged by their relative numbers as well as the elaboration of their processes (Figure 1C).

**Figure 2:**
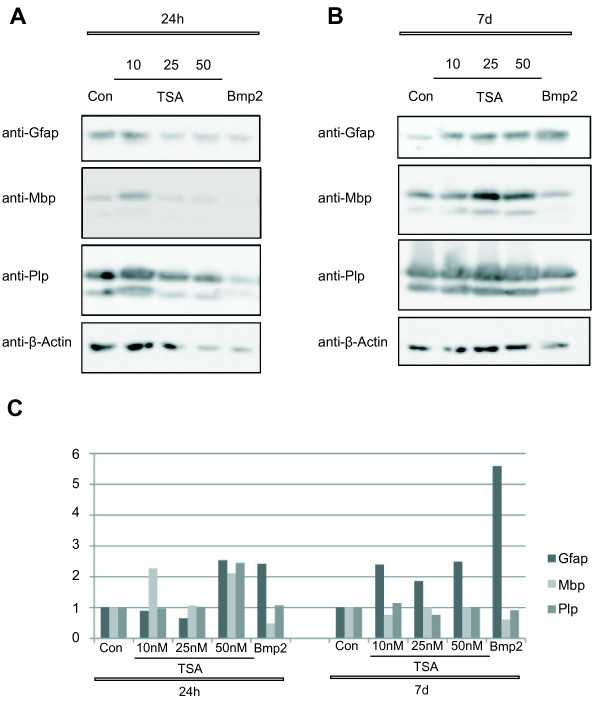
** Western Blot of linage specific markers.** Proteins were extracted from TSA (10, 25, 50nM) and BMP2 (10 ng/μl) treated neurosphere cultures 24 h (**A**).and 7 days (**B**) after treatment. Western Blot for Plp, Mbp, and Gfap was performed. β-Actin was used as control. Western Blots were quantified and normalized to β-Actin (**C**). The results shown are one example of three repetitions.

In addition western blot analysis of astrocyte and oligodendrocyte specific proteins 24h (h) and 7 days after treatment with TSA or BMP2 were performed (Figure 2). The expression of the astrocyte marker protein GFAP significantly increased upon TSA and BMP2 treatment. Whereas the protein levels of GFAP was rather unchanged 24 h after treatment with TSA and BMP2 (Figure 2A), a strong increase of GFAP could be detected 7 days after treatment (Figure 2B), indicating that the treatment with TSA and BMP2 led to an increase in astrogliogenesis during differentiation of neurosphere cultures. The oligodendrocyte markers Plp (proteolipid protein) and Mbp (myelin basic protein) [30] were less clearly regulated on the protein level at both time points, but a small decrease of both markers could be detected 7 days after treatment (Figure 2).

### Microarray analysis of differentiating neurosphere cultures

**Figure 3:**
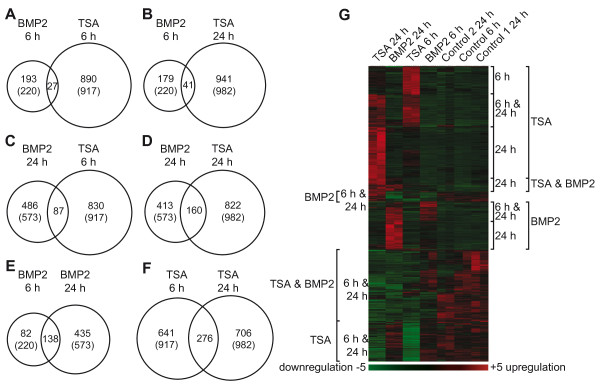
** Gene expression profiling of BMP2 and TSA treated neurosphere cultures.** Differentially expressed genes (relative expression > 2-fold and absolute difference >100, comparing treated and control sample, confidence > 90%) are presented as Venn-diagrams (**B**-**G**) and hierarchical cluster (**H**). Venn diagrams present the number of genes regulated upon TSA and BMP2 treatment after 6 h and 24 h. Numbers of regulated genes are indicated in the diagram, the total number of regulated genes for each treatment is put in parentheses. Intersection of regulated genes between TSA 6 h and BMP2 6 h (**B**), TSA 24 h and BMP2 6 h (**C**), TSA 6 h and BMP2 24 h (**D**), TSA 24 h and BMP2 24 h (**E**), BMP2 6 h and BMP2 24 h (**F**) and TSA 6 h and TSA 24 h (**G**) are illustrated. Differentially expressed genes are clustered in a hierarchical cluster analysis. Red and green depict at least two-fold increase or decrease in expression, respectively. Clusters including upregulated genes are labeled at the right side of the cluster plot. Clusters, grouping genes downregulated after treatment, are labeled on the left side (**H**). Genes from individual main sub-cluster of hierarchical cluster analysis were functional annotated using DAVID Database.

RNA samples and protein lysates were prepared 6, 12, and 24 h after treatment. We performed gene expression profiling from cells treated for 6 h and 24 h using Affymetrix GenChip 420 2.0. The raw data was analyzed using dChip (DNA-chip analyzer) software [31]. Genes were considered to be significantly regulated if their expression had changed more than two-fold and had exceeded a minimal absolute difference of 100 comparing treated and mock-treated cells with a confidence greater than 90%. Using these conditions 220 genes exhibited a differential expression in BMP2-treated cells after 6 h, and 573 genes were differentially regulated after 24 h (Figure 3A-F). TSA treatment led to 917 differentially-expressed genes after 6 h and 982 after 24 h treatment (Figure 3A-F). The top 25 genes regulated after TSA or BMP2 treatment are listed in the Additional file 1: Table S1–S4.

To identify an overlap of regulated genes within TSA and BMP2 treatments, two-set Venn analyses were performed, intersecting TSA 6 h, TSA 24 h, BMP2 6 h and BMP2 24 h experimental sets, respectively. The intersection between two experimental sets is shown in individual Venn diagrams (Figure 3A-F). The numbers of regulated genes for each treatment condition are depicted in the diagrams, while individual genes within the intersection are listed in the Additional file 1: Table S5–S10. Comparing these two-set Venn diagrams, it could be observed that the majority of regulated genes was unique for one treatment; only a smaller number of genes was located within the intersections between the two experimental sets. The largest intersection of regulated genes was detected between TSA 6 h and TSA 24 h (Figure 3F). The intersection between the BMP2 6 h and 24 h experiments was marginally smaller; however more than half of the genes regulated after 6 h overlap with genes regulated after 24 h (Figure 3E). Comparing the Venn analyses of TSA- and BMP2-treated samples at the two different time points an increased number of co-regulated genes could be detected from 6 h to 24 h (Figure 3A-D). Whereas only 27 genes were regulated in both BMP2 6 h and TSA 6 h (Figure 3A), the number of regulated genes in BMP2 24 h and TSA 24 h experimental sets increased 6-fold (Figure 3D). This increased number of regulated genes in the intersection of TSA and BMP2 treated sample after 24 h mainly resulted from an increase of regulated genes in the BMP2 24 h sample, even if the number of regulated genes in BMP2 24 h experiment is only 2.6-fold higher than in BMP2 6 h experiment (Figure 3A, 3D).

In addition to the two-set Venn analyses, the overlap of genes regulated in all four sets of experiment and in three out of four sets was additionally analyzed. A summary of these genes is listed in Table 1, indicating their fold-change in each treatment. Remarkably, only eight genes were significantly altered after the treatment with BMP2 and TSA at both time points: *Gpr17* (G protein-coupled receptor 17), *Lims2* (LIM and senescent cell antigen like domains 2), *Bcas1* (breast carcinoma amplified sequence 1), *Ptpre* (protein tyrosine phosphatase, receptor type, E), *Afap1l2* (actin filament associated protein 1-like 2), *Dll3* (delta-like 3), *G0s2* (G0/G1 switch gene 2), *Gpd1* (glycerol-3-phosphate dehydrogenase1). 65 genes were regulated in at least three out of four experimental sets. Most of these genes were regulated in the same direction when treated with BMP2 or TSA, and only a few genes exhibited an opposed expression. *Smad7* (MAD homolog 7 (Drosophila)), *Papss2* (3′-phosphoadenosine 5′-phosphosulfate synthase 2), *Fam19a2* (family with sequence similarity 19, member A2), *Cadps* (Ca^2+^-dependent secretion activator), *Car8* (carbonic anhydrase 8) and *Efhd1* (EF hand domain containing 1) are examples for an opposed regulation of expression comparing BMP2- and TSA-treated samples. *Smad7*, *Papss2*, *Fam19a2*, and *Cadps* expression was suppressed after TSA treatment but induced after treatment with BMP2, whereas *Car8* and *Efhd1* expression was regulated in a reverse fashion (Table 1).

**Table 1:** Genes significantly regulated upon TSA and BMP2 treatment

**Gene name**	**Gene name symbol**	**BMP2**		**TSA**	
		**6 h**	**24 h**	**6 h**	**24 h**
G protein-coupled receptor 17	**Gpr17**	−3.13	−30.90	−6.80	−80.21
ectonucleotide pyrophosphatase/phosphodiesterase 6	Enpp6	−1.99	−23.08	−3.93	−30.02
LIM and senescent cell antigen like domains 2	**Lims2**	−2.80	−24.72	−4.61	−27.27
plexin B3	Plxnb3	−1.12	−8.69	−4.61	−26.64
serine/arginine-rich protein specific kinase 3	Srpk3	−1.22	−7.54	−6.01	−26.60
galactose-3-O-sulfotransferase 1	Gal3st1	−1.01	−8.92	−4.96	−18.20
breast carcinoma amplified sequence 1	**Bcas1**	−2.80	−24.38	−9.53	−17.36
bone morphogenetic protein 4	Bmp4	−9.01	−34.17	−3.25	−15.47
S100 protein. beta polypeptide. neural	S100b	1.19	−4.14	−3.54	−14.71
ELOVL family member 7. elongation of long chain fatty acids	Elovl7	−1.21	−6.57	−3.33	−14.34
breast carcinoma amplified sequence 1	Bcas1	−1.55	−11.02	−2.93	−13.89
RAB33A. member of RAS oncogene family	Rab33a	−1.50	−9.41	−7.10	−13.66
SRY-box containing gene 10	Sox10	−1.28	−6.73	−2.81	−13.51
protein kinase C. theta	Prkcq	−2.52	−4.70	−11.82	−12.45
tubulin. beta 4	Tubb4	−1.68	−5.93	−4.74	−12.31
Down syndrome cell adhesion molecule	Dscam	−2.02	−4.85	−3.70	−11.67
oligodendrocyte myelin glycoprotein	Omg	−1.38	−2.61	−11.48	−9.86
four and a half LIM domains 2	Fhl2	−1.11	−11.15	−6.79	−9.48
glycerol-3-phosphate dehydrogenase 1	**Gpd1**	−2.93	−8.02	−2.50	−7.87
chimerin (chimaerin) 2	Chn2	−1.41	−6.46	−5.09	−7.68
protein tyrosine phosphatase. receptor type. E	**Ptpre**	−18.37	−16.45	−2.53	−7.19
Down syndrome cell adhesion molecule-like 1	Dscaml1	−3.25	−2.48	−3.19	−5.91
phosphatase and actin regulator 3	Phactr3	−3.18	−6.73	−1.86	−5.81
MAD homolog 7 (Drosophila)	Smad7	4.51	6.86	−3.24	−5.69
family with sequence similarity 19. member A2	Fam19a2	3.13	1.03	−3.10	−5.38
leucine rich repeat transmembrane neuronal 1	Lrrtm1	−1.90	−4.49	−7.54	−5.20
solute carrier family 24 member 3	Slc24a3	−2.44	−1.87	−3.79	−4.96
delta-like 3 (Drosophila)	**Dll3**	−4.14	−3.64	−3.32	−4.49
shroom family member 2	Shroom2	−1.39	−2.78	−3.69	−4.46
WAS/WASL interacting protein family member 1	Wipf1	−1.20	−2.68	−3.89	−4.20
actin filament associated protein 1-like 2	**Afap1l2**	−3.47	−3.68	−4.84	−4.16
seizure related 6 homolog like	Sez6l	−3.30	−2.91	−2.92	−4.16
proline rich region 18	Prr18	−1.71	−3.35	−6.74	−4.09
striatin. calmodulin binding protein	Strn	−1.55	−2.35	−2.94	−4.07
G0/G1 switch gene 2	**G0s2**	−3.89	−5.35	−6.74	−4.02
cytoplasmic FMR1 interacting protein 2	Cyfip2	−2.12	−4.22	−2.53	−3.79
platelet derived growth factor alpha	Pdgfa	−2.27	−5.57	−4.54	−3.59
3'-phosphoadenosine 5'-phosphosulfate synthase 2	Papss2	6.90	10.70	−2.50	−3.34
dedicator of cytokinesis 9	Dock9	−1.86	−5.59	−2.63	−3.28
zinc finger protein 365	Zfp365	−3.75	−5.30	−2.43	−3.23
ring finger protein 122	Rnf122	−2.05	−2.76	−2.63	−2.92
SH3-domain binding protein 4	Sh3bp4	−3.26	−4.35	−1.82	−2.79
erythrocyte protein band 4.1	Epb4.1	−2.43	−3.67	−1.30	−2.55
TNF receptor associated factor 4	Traf4	−3.54	−4.89	−2.20	−2.45
myelocytomatosis oncogene	Myc	−2.37	−4.17	−3.82	−1.70
Ca2 + −dependent secretion activator	Cadps	2.90	4.96	−2.52	−1.39
GS homeobox 1	Gsx1	−2.52	−5.17	−4.86	−1.20
leucine rich repeat and fibronectin type III. extracellular 1	Elfn1	−4.08	−6.57	−2.81	−1.06
myosin regulatory light chain interacting protein	Mylip	7.66	4.10	3.47	1.55
tumor necrosis factor receptor superfamily. member 12a	Tnfrsf12a	9.04	10.10	7.39	1.87
carbonic anhydrase 8	Car8	−2.65	−6.46	2.65	2.06
epidermal growth factor receptor	Egfr	−4.15	−7.93	−1.95	2.73
BMP and activin membrane-bound inhibitor. homolog (Xenopus laevis)	Bambi	3.46	3.60	2.61	3.41
follistatin	Fst	16.56	19.63	1.55	4.18
Rho GTPase activating protein 29	Arhgap29	2.69	8.01	5.84	4.35
DIRAS family. GTP-binding RAS-like 2	Diras2	12.12	99.94	1.09	4.51
aquaporin 11	Aqp11	1.66	5.08	3.24	4.61
Kruppel-like factor 4 (gut)	Klf4	5.39	13.51	7.94	4.74
regulator of G-protein signalling 10	Rgs10	1.01	3.25	3.20	6.39
EF hand domain containing 1	Efhd1	−1.52	−5.45	3.22	6.77
KDEL (Lys-Asp-Glu-Leu) endoplasmic reticulum protein retention eceptor 3	Kdelr3	1.62	7.32	11.15	6.87
zinc finger. CCHC domain containing 12	Zcchc12	1.95	4.24	8.50	9.35
family with sequence similarity 70. member A	Fam70a	7.28	13.35	2.99	16.08
predicted gene 98	Gm98	−2.39	−13.78	−4.13	−20.43

Genes significantly regulated upon all treatments (printed in bold) and three out of four treatments are summarized.

In accordance with the results from the two-set Venn analysis, the number of co-regulated genes was increased when the BMP2 24 h time point was included in the intersection analysis (Table 1). However, among these genes, the expression of only a few was significantly stronger regulated after 24 h than after 6 h of both TSA and BMP2 treatment. Especially, the expression of *Gpr17*, *Lims2*, *Bcas1*, *BMP4*, *Enpp6* (ectonucleotide pyrophosphatase/phosphodiesterase 6) and *Gm98* (predicted gene 98) was significantly reduced in 24 h compared to 6 h experiments. It should be mentioned that, among those genes regulated by BMP2 6 h and 24 h and TSA 24 h, several genes known to be involved in BMP2/4 signaling, like *Bmp4*, *Smad7*, *Fst* (Follistatin) and *Bambi* (BMP and activin membrane-bound inhibitor, homolog (*Xenopus laevis*)) were detected.

We also performed hierarchical clustering of the microarray data using the clustering option of dChip [31] to illustrate the overall relationship between regulated genes (Figure 3G). All genes regulated in at least one of the analyzed conditions were included using the same stringent criteria as above (twofold change; euclidean distance 100). The clustering led to two major clusters, one including genes upregulated, the other including genes downregulated upon either treatment. Genes upregulated after each treatment were further divided into three sub-clusters, grouping genes upregulated after treatment with (i) BMP2 or (ii) TSA alone or (iii) both BMP2 and TSA. Each sub-cluster could be subdivided into smaller groups of genes that represent individual time points. Within the cluster of downregulated genes, also three sub-clusters could be distinguished, containing genes downregulated after treatment with (i) BMP2 or (ii) TSA alone (clustered in the middle of Figure 3G) and (iii) downregulated after both treatments.

To investigate the specific biological functions of co-regulated groups of genes, we used the DAVID Database (Database for Annotation, Visualization and Integrated Discovery) for functional annotation of clustered genes [32,33]. Functional annotation clustering allows the classification of regulated genes according to their functional relevance. Each of the six sub-clusters obtained in the hierarchical clustering was independently annotated. An overview of the various functional categories for the six sub-clusters is shown in Table 2, Gene Ontology annotations of individual clusters can be found in Additional file 1: Tables S11-S21. Strong differences in the functional categories arose upon comparison of up- and downregulated gene clusters. Within the gene cluster including genes upregulated after TSA treatment, functional categories like antigen processing, metabolism, cell membrane and cell adhesion were enriched (Additional file 1: Table S11-S14), the cluster of downregulated genes included functional categories related to chromosome organization, transcriptional processes, metabolism, and posttranslational processes (Additional file 1: Table S15). In the case of BMP2 treatment, the gene cluster of upregulated genes was enriched for functional categories associated with cell communication, cell membrane, extracellular matrix, differentiation and development (Additional file 1: Table S18-S20). Genes downregulated after BMP2 treatment were enriched in the functional categories related to cell communication and signal transduction (Additional file 1: Table S21). The functional annotation of the sub-cluster containing genes upregulated after both treatments showed an enrichment of categories related to extracellular matrix and cell adhesion, whereas the sub-cluster of downregulated genes comprised categories related to differentiation and development (Additional file 1: Table S16-S17). As well from the list of individual genes as from the functional cluster analysis it was apparent that BMP2 and TSA treatment resulted in independent gene profiles. While TSA treatment mainly led to a regulation of transcriptional processes, BMP2 treatment rather resulted in a regulation of signal transduction processes. Even though both treatments primarily led to a different expression of genes, the downregulation of certain genes seems to reflect the similar phenotype which we had observed in both TSA- and BMP2-treated neurosphere cultures. While only a few primary target genes of TSA and BMP2 were clustered within the sub-clusters containing genes regulated after both treatments, it is obvious that a variety of genes involved in neural development were present, such as the oligodendrocyteproteins *Mag* (myelin-associated glyprotein), *Mal* (myelin and lymphocyte protein), *Mog* (myelin oligodendrocyte glycoprotein), *Omg* (oligodendrocyte myelin glycoprotein), *Mbp* (myelin basic protein), and *Mobp* (myelin-associated oligodendrocytic basic protein), which were downregulated in one or both treatments.

**Table 2:** Overview of the functional categories for the six sub-clusters of hierarchical clustering

**Gene cluster: TSA up-regulated**		**Gene cluster: TSA down-regulated**	
·	antigen processing	·	chromosome organization
·	metabolism	·	nucleus
·	membrane, adhesion	·	transcription
·	cell part	·	metabolism
		·	protein modification
**Gene cluster: Bmp2 up-regulated**		**Gene cluster: Bmp2 down-regulated**	
·	cell communication	·	cell communication
·	differentiation, development	·	signal transduction
·	membrane, extracellular matrix		
**Gene cluster: Bmp2, TSA up-regulated**		**Gene cluster: Bmp2, TSA down-regulated**	
·	adhesion, extracellular matrix	·	differentiation, development
		·	neurogenesis

**Figure 4:**
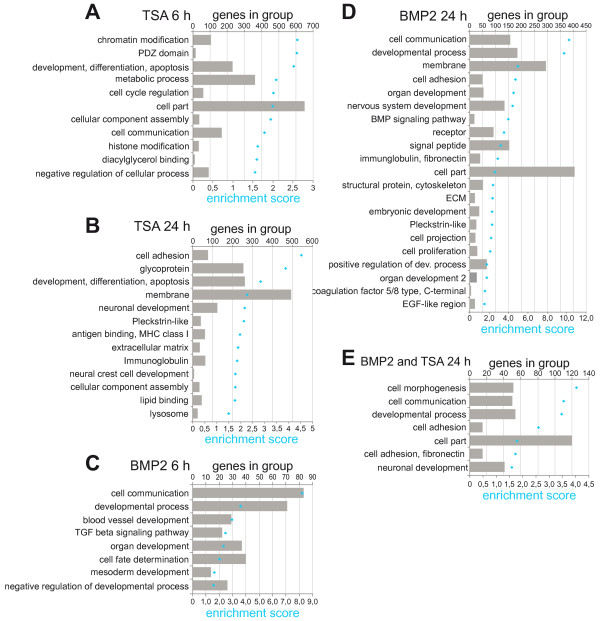
** Functional annotation clustering of BMP2 and TSA treated neurosphere cultures at different time points.** Differentially expressed genes (relative expression >2-fold and absolute difference >100. comparing treated and control sample, confidence >90%) are functionally clustered using DAVID Database. Functional groups with an enrichment score >1.5 are exhibited. The enrichment score is the geometric mean (in -log scale) of member's p-values in a corresponding annotation cluster. The number of genes in each cluster is represented as bars, the corresponding enrichment scores are illustrated as dots. Functional annotation of genes altered in individual treatments with TSA after 6 h (**A**) and 24 h (**B**), BMP2 after 6 h (**C**) and 24 h (**D**) as well as of intersectional genes regulated after treatment with BMP2 24 h and TSA 24 h (**E**) are displayed.

Since the functional annotation clustering did not disclose an enrichment of direct target genes of TSA or BMP2, and because we detected the strongest overlap of regulated genes between TSA and BMP2 treatment after 24 h, we decided to perform an additional DAVID analysis including genes regulated significantly after different times of treatment. Figure 4 summarizes the clustered functional categories obtained from TSA 6 h, TSA 24 h, BMP2 6 h or BMP2 24 h experiment; only such functional annotation clusters are shown that possessed a significant enrichment score of 1.5. Gene Ontology annotations for the clusters can be found in (Additional file 1: Table S22-S26). The functional categories obtained after BMP2 6 h treatment included primarily genes with functions related to developmental processes (Figure 4C). In contrast, after 24 h treatment a more diverse set of functional categories was enriched, possessing functions in plasma membrane, cell adhesion, antigen-presenting and developmental processes (Figure 4D). After TSA 6 h treatment, categories were enriched which contained genes with functions in histone modification, chromatin organization, transcription regulation and cell cycle control (Figure 4A). Similar to the 24 h BMP2 treatment, the 24 h TSA treatment also showed a more diverse set of functional categories (Figure 4B). Interestingly, these categories resembled the categories enriched after BMP2 24 h treatment. This functional overlap is also reflected in functional annotation clustering of genes regulated in both BMP2 24 h and TSA 24 h samples. Clusters with genes involved in functions in plasma membrane, cell adhesion, cell communication, as well as genes involved in developmental processes were enriched (Figure 4E). This suggests that genes regulated after 6 h directly reflected the well-established activity on gene regulation mediated by histone deacetylase inhibition, but that after 24 h already a secondary biological effect may have been observed.

### Validation of the microarray data with mRNA expression analysis

**Figure 5:**
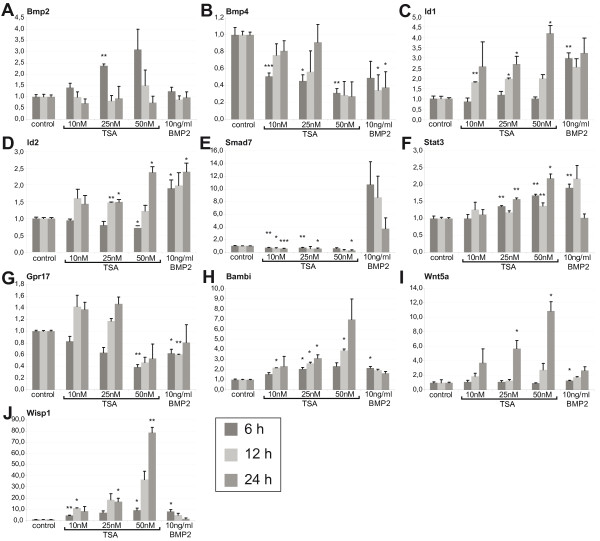
** Quantitative real-time PCR.** Total RNA was extracted from TSA and BMP2 treated neurosphere cultures. Neurosphere cultures were cultured for 7 days and subsequently dissociated and plated out in monolayers. Cells were treated with TSA (10, 25, 50nM) or 10 ng/μl BMP2 from 1.5-2.5 days after plating. Total RNA was collected 6, 12 or 24 h upon treatment. Reverse-transcript cDNA was analyzed using primer recognizing the following genes: *Bmp2* (**A**), *Bmp4* (**B**), *Id1* (**C**), *Id2* (**D**), *Smad7* (**E**), *Stat3* (**F**). *Gpr17* (**G**), *Bambi* (**H**), *Wnt5a* (**I**), *Wisp1* (**J**) and Primer recognizing *beta-Actin* and *Gapdh* were used for normalization. Shown values represent the mean (+/− SEM) of three individual experiments (6 h and 24 h) or two individual experiments (12 h). * = p < 0.05, ** = p < 0.01, *** = p < 0.001, Student’s *T*-test.

For validation of the microarray data, we selected several genes and performed quantitative RT-PCR. *Gpr17* (Figure 5G), *Bambi* (Figure 5H), *Smad7* (Figure 5E) and *Bmp4* (Figure 5B) were chosen from the lists of genes regulated in both TSA and BMP2 treatment. In order to obtain a more detailed view of the regulatory response, one additional time point and two additional TSA concentrations were used (10, 25, or 50nM TSA or 10 ng/ml BMP2; 6 h, 12 h or 24 h). All selected genes showed consistent expression patterns in RT-PCR and the microarray experiments, although fold-changes determined in the microarray analysis and the quantitative RT-PCR differed significantly. In addition to *BambiSmad7* and *Bmp4*, known to be involved in BMP signaling, we decided to analyze the expression of *Bmp2* (Figure 5A) and the BMP target genes *Id1* (Figure 5C) and *Id2* (Figure 5D). While *Bmp4* was downregulated upon TSA treatment (Figure 5B), the expression of *Bmp2* was significantly upregulated in a concentration dependent manner after 6 h, but not after 12 and 24 h (Figure 5A). Surprisingly, *Id1* and *Id2* expression was downregulated at 6 h, but increased after 12 h, resulting in a similar level of expression compared with BMP2-treated cells after 24 h, suggesting a partial BMP signaling-independent effect on Id expression. We furthermore investigated *Stat3* (signal transducer and activator of transcription 3) (Figure 5F), known to be an upstream regulator of BMP expression [34] but also to co-regulate astrocyte specific genes through the formation of a STAT3-p300-Smad complex [35]. We also decided to analyze *Wnt5a* (wingless-related MMTV integration site 5A) (Figure 5I) and *Wisp1* (WNT1 inducible signaling pathway protein 1) (Figure 5J), both of which are involved in Wnt signaling and are known to act upstream of BMP signaling [36]. *Stat3* (Figure 5F), as well as *Wnt5a* (Figure 5I) and *Wisp1* (Figure 5J), were significantly upregulated upon TSA treatment in a time and concentration dependent manner.

### Validation of the microarray data with protein analysis

**Figure 6:**
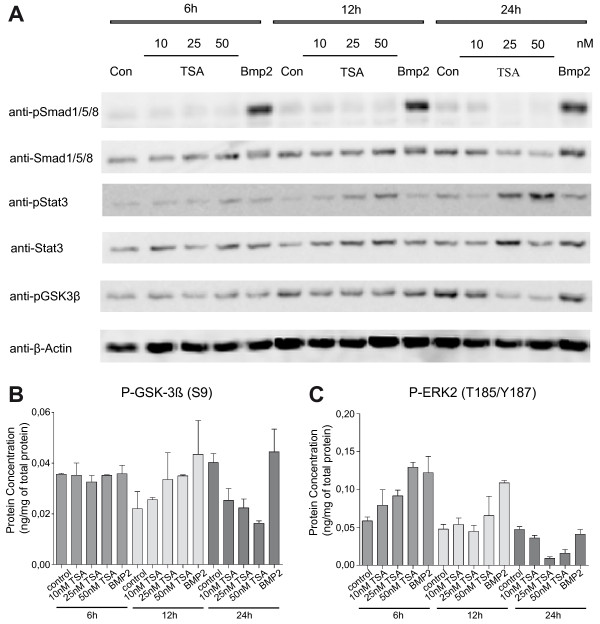
** Western Blot and ELISA Microarray.** Proteins were extracted from TSA (10, 25, 50nM) and BMP2 (10 ng/μl) treated neurosphere cultures 6, 12 or 24 h after treatment. Western Blot for pSmad1/5/8, Smad1/5/8, pStat3, Stat3, pGsk3-beta was performed. Beta-Actin was used as control (**A**). Phosphorylated proteins were quantified using an ArrayTube™ (Alere Technologies, Jena, Germany) based sandwich ELISA microarray. Concentration of pGsk3-beta (**B**) and pErk2 (**C**) in relation to total protein content is presented.

Based upon the upregulation of *Stat3* mRNA expression (Figure 5F), and its known role in BMP2-triggered astrogliogenesis, we performed Western blot analysis of Stat3 and other proteins known to be involved in signaling during astrogliogenesis. We investigated the phosphorylation of Smad1/5/8, known mediators of BMP signaling, Stat3, and Gsk3-beta, a signaling protein in the canonical Wnt signaling pathway (Figure 6). Smad1/5/8 was phosphorylated in the BMP2 treated samples after 6, 12 and 24 h, but TSA treatment did not lead to Smad1/5/8 phosphorylation (Figure 6A). In contrast, pStat3 was strongly induced after TSA treatment (25nM and 50nM), showing an increase from 6 h to 24 h, while BMP2 treatment did not induce Stat3 phosphorylation (Figure 6A). Treatment with TSA led to a strong reduction of Gsk3-beta phosphorylation after 24 h, whereas almost no change could be detected after 6 h and phosphorylation was rather increased after 12 h (Figure 6B). The concentration of pGsk3-beta was quantified using an ArrayTube™ (Alere Technologies, Jena, Germany) based sandwich ELISA microarray. Interestingly, the sandwich ELISA microarray disclosed a clear regulation of Erk2 phosphorylation upon both BMP2 and TSA treatment (Figure 6C). At the 6 h and 12 h time point pErk2 was induced in a concentration dependent manner after TSA treatment, but also after BMP2 treatment. After 24 h of treatment the pErk2 signal clearly decreased, which suggested that pErk2 is involved in the early signaling following BMP2 and TSA treatment.

## Discussion

We previously demonstrated that treatment of neuronal precursor cells derived from the ganglionic eminences (GE) with BMP2 or TSA resulted in a reduction in the generation of neurons and oligodendrocytes and in an increase in the production of astrocytes [27]. In this study, we performed gene expression profiling upon cultures treated with either BMP2 or TSA in order to identify common genes and signaling pathways regulating the differentiation of GE neural precursor cells. The fact that treatment with BMP2 or TSA resulted in identical cell fates was reflected in the gene expression data by a significant overlap of regulated genes. Comparing the 6 h and 24 h experiments, it became obvious that the overlap of regulated genes between both treatments increased with the duration of time. After 6 h the gene expression profile between BMP2 and TSA treatment differed significantly. Short treatment with TSA resulted in regulation of genes related to histone/chromatin modification, drug response, and fundamental cellular functions, whereas BMP2 treatment led to an early regulation of developmental processes via activation of BMP signaling. This difference in the early response confirms the specificity of both treatments. Treatment with the small molecule inhibitor TSA elicits an induction of stress response genes, including heat shock proteins (*Hspa1b, Hspa1a* (heat shock protein 1A/B)) [37], oxidative stress (*Gstt3* (glutathione S-transferase, theta 3), *Txnip* (thioredoxin interacting protein)) [38,39] and damage response genes (Ier3 (immediate early response 3), *Pmaip1* (phorbol-12-myristate-13-acetate-induced protein 1)) [40]. A dominant effect of TSA treatment is the regulation of chromatin organization and remodeling genes, which are significantly enriched (p-value <10^-6^). The distribution of GO groups regulated by TSA is comparable to published data [41].

After 24 h, both treatments resulted in a more similar expression profile, not just by an overlap of individual genes but also through the alteration of several groups of genes regulating cell communication, cell adhesion and developmental processes. As shown previously, a 24hour treatment with TSA just before bFGF withdrawal was sufficient to promote astrogliogenesis and inhibit the birth of neurons and oligodendrocytes in GE-derived precursor cultures [27]. Together with the results from gene expression profiling it can be assumed that a short treatment with TSA stimulates these cell fate decisions through epigenetic modification that lead to the up- and downregulation of the corresponding developmental genes, though it is also possible that the effect does not occur at the transcriptional level, but rather through acetylation of cytoplasmic or nuclear non-histone proteins. The stronger correlation in the 24 h gene expression data set between TSA- and BMP2-treated cultures reveals that even if the early gene expression response to the treatment differs dramatically, similar downstream processes are induced, resulting in a comparable cell fate phenotype.

BMPs can enhance neurogenesis or gliogenesis depending upon the developmental stage or brain localization. During mid-gestation BMPs are neurogenic [9,10], whereas in late prenatal and postnatal stages they are astrogliogenic [5,6]. The data from gene expression profiling revealed however a surprising downregulation of BMP signaling genes after TSA or BMP2 treatment. *Bmp4* and the receptors *Bmpr1a* and *Bmpr1b* were significantly downregulated in response to TSA, whereas BMP signaling inhibitors, like *BambiCtgf* (connective tissue growth factor) and *Fst*, were significantly upregulated. Only the BMP signaling inhibitor *Smad7* was significantly downregulated after TSA treatment. This indicates that both TSA and BMP2 initiate both a direct, positive response activating the downstream BMP signaling pathway as well as a subsequent negative feedback loop that results in induction of BMP signaling inhibitors and downregulation of BMP4 and its receptors. This clear inactivation of further BMP signaling could reflect the transition between differentiation states, which requires changes in sensitivity to BMP signals. Sensitivity to growth factors as well as the duration of signals plays an important role for BMP- and TGF-beta family signaling in development. Established mechanisms include selective expression and degradation of receptors and in particular a range of mechanisms to control the duration of the signal of activated regulatory Smads (Smad1/5/8, Smad2/3) through negative feedback mechanisms, including expression of inhibitor Smads (Smad6 and Smad7) and dephosphorylation and degradation of regulatory Smads [42].

Interestingly, our gene expression profiling did not show an increase in astrocyte-specific genes. Classic markers known to be upregulated during astrocyte differentiation were either not regulated (e.g. *Gfap* (Glial fibrillary acidic protein)), or were downregulated (e.g. S100ß (S100 protein, beta polypeptide)). This prompted us to look more closely at the array data and indeed we were able to identify two transcription factors, *Id1* and *Id2*, whose expression levels did not significantly change in the array studies but whose expression was documented to significantly increase upon either BMP2 or TSA treatment. BMP2 has previously been shown to cause upregulation of *Id1* and *Id2*[43], and forced expression of either gene can inhibit neurogenesis in telencephalic cultures [8], suggesting that these two factors play a role in the BMP-promoted switch from neurogenesis to astrogliogenesis. In addition, we could demonstrate significant increases in the mRNA and protein levels of Stat3 and also in its phosphorylated, transcriptionally active form. This is of particular relevance for astrogliogenesis as Stat3 has been shown to functionally interact with the BMP2-responsive transcription factor Smad1/5/8 at the p300 transcriptional coactivator and thereby synergistically promote astrogliogenesis [35]. How TSA promotes an increase in Stat3 levels is unclear at this point, but we have uncovered evidence that the acetylation of Stat3 is regulated by TSA-mediated HDAC inhibition (data not shown).The transient activation of Erk2 in response to BMP2 and TSA treatment could play a role in the control of the duration of activated Smad1/5/8 signals. Erk2, but also other kinases, including Gsk3-beta, are involved in the control of Smad signals through Smad linker phosphorylation [44,45]. Phosphorylation of the linker region by Erk2 and Gsk3-beta targets regulatory Smads for ubiquitinylation and proteasomal degradation [44,45]. The observed activation of Erk2 should lead to a more rapid degradation of activated Smads, which can be further modulated by Gsk3-beta. Thus, induction of Erk2 by phosphorylation would contribute to termination of BMP signals [44,45].

Analysis of the genes upregulated in response to TSA and BMP2 treatment revealed several genes known to be expressed in neurons. Most of these genes are not markers or regulators of basic neurogenesis, but are rather involved in maturation processes or establishment of the neuronal network, such as neurite outgrowth, axon guidance and synapse maturation and function. The fact that we see an upregulation of these genes can be possibly explained by the developmental age of the cultures, which were derived from E15.5 GE. At this time point neurogenesis has reached its peak, before radial glia cells in GE start to generate astrocytes [46,47]. It is possible that both TSA and BMP2 are upregulating the expression of functional neuronal genes in those precursors that have already committed to the neuronal fate or have already been born as neurons. The cultures in our experiments were treated at 2.5 DIV, and a small amount of neurogenesis has already occurred at this time point [27]. In addition, it is known that markers of maturing neurons already begin to be expressed by neuronal progenitors [48,49].

TSA and BMP2 treatment results in a drastic downregulation of genes known to be specific for oligodendrocytes, such as *Sox10* (SRY-box containing gene 10) and *Nkx2-2* (NK2 transcription factor related, locus 2), a variety of genes involved in myelinization *MagMalMogOmgMbpMobpGm98*, and other genes known to be highly expressed in oligodendrocytes, such as *Gpr17Bcas1*, and *Enpp6*[50]. The fact that genes involved in myelinization were strongly regulated explains the appearance of membrane-related GO terms in the functional annotation clustering. The downregulation of oligodendrocyte specific genes in our experiments is in accordance with a reduction of oligodendrocytes that was observed by ourselves [27] and others [19]. Many of these oligodendrocyte specific genes were not only significantly down-regulated upon TSA treatment but also after BMP2 treatment, especially after 24 h. This also corresponds with previous reports showing that BMPs promote the production of astroglia while inhibiting oligodendrocyte differentiation [7]. The fact that treatment with BMP2 and TSA downregulates oligodendrocyte specific genes seems to be a common feature of both compounds, but it still needs to be clarified if the demonstrated effect is due to the same regulatory mechanism. Upregulation of *Wnt5aWisp1*, and other genes from Wnt signaling in our experiments could give a certain indication that the regulatory mechanism could be related in both cases. Wnt signaling leads to the suppression of oligodendrocyte differentiation and promotes neuronal and astroglial differentiation [51]. The connection between BMP and Wnt signaling [36] as well as between HDACs and Wnt signaling [21] had been shown to be important for astroglial and oligodendroglial lineage commitment, and it will be of great interest to examine whether HDACs and BMPs share a common pathway in the regulation of oligodendrocyte differentiation, as we have shown for astrocyte differentiation in this work.

## Conclusions

In this study we have delineated at the genomic transcriptome level the responses to two different compounds that we and others have shown to lead to similar biological outcomes in the differentiation of neural progenitor cells to neurons, astrocytes and oligodendrocytes in the embryonic forebrain. Interestingly, the range of responses to BMP2 and to the global HDAC inhibitor TSA were dramatically different, with BMP leading to an upregulation of genes involved in cell-cell communication and developmental processes while TSA resulted in an upregulation of genes involved in chromatin modification and transcription. Surprisingly, the biological convergence of the genomic responses could not be reduced to canonical BMP signaling through Smad1/5/8 activation, rather HDAC inhibition and BMP2 signaling converge through Stat3 and Smad1/5/8-mediated signaling and Id1 activation which increases astrogliogenesis from neural stem cells. This result explains the similar outcomes of HDAC inhibition and BMP with respect to astrogliogenesis, and the microarray profiling also suggests new pathways, for example Wnt signaling, which may be of further relevance for the interaction between these two developmentally-crucial protein families.

## Methods

### Mouse lines

All animal experiments were conducted in compliance with the regulations of the state of Baden-Württemberg, Germany. We employed C57BL/6 J mice (Charles River, Sulzfeld, Germany).

### Neurosphere cultures

Neurosphere (NS) cultures were prepared from E15.5 GE essentially as described [27]; full protocol described in [29]. Embryos were dissected on ice in PBS and decapitated. The brain was removed, the hemispheres separated, and the lateral and medial ganglionic eminences removed with fine forceps. GE cells were mechanically dissociated with a fire-polished Pasteur pipette and plated out in cell culture flasks with 100,000 cells per milliliter (ml) in NS Medium (F12/DMEM (1:1) with B27 supplement (Invitrogen), penicillin/streptomycin (100U/ml, Invitrogen), human EGF (20 ng/ml; Sigma) and human bFGF (10 ng/ml; R&D Systems, Wiesbaden, Germany). NS were incubated in suspension at 37°C, 5% CO_2_ for 1 week and fed on the 5^th^ day with an equal volume of NS medium. For differentiation, 7 day-old NS were collected into 50-ml tubes and centrifuged for 3 minutes at 100xg. The NS were mechanically dissociated using a fire-polished Pasteur pipette and plated out with 150,000 cells per cm^2^ in petri dishes (pre-plated with 200 mg/ml polyornithine) in NS medium without EGF and with 1% fetal calf serum (FCS, Invitrogen), a medium that supports the differentiation of both neurons and astrocytes. After 2.5 days of incubation the medium was changed to NS medium without bFGF and EGF but with 1% FCS. The following pharmacological reagents were added in different experiments: 10, 25 or 50nM trichostatin A (Sigma, Calbiochem), recombinant BMP2 (10 ng/ml), 6 h, 24 h.

### Immunocytofluorescence

Neurospheres were cultured as described above, treated with 50nM trichostatin A, recombinant BMP2 (10 ng/ml), or both reagents for 24 hours before bFGF withdrawal, cultured for another 4.5 days, and fixed with 4% PFA for 10 min. Cultures were stained as described [27] with the following antibodies: TuJ1 (Covance), O4 (kind gift of Prof. J. Trotter, Mainz, Germany), anti-GFAP (DAKO), and DAPI (Sigma-Aldrich). Confocal analysis was performed on a Nikon A1Rsi microscope (Nikon Imaging Center, University of Heidelberg).

### RNA Isolation

Total RNA was isolated from neurosphere culture 6, 12, and 24hours (h) after treatment using RNeasy Mini Kit (Qiagen) according to the manufacturer’s instructions. RNA quality was examined by agarose gel electrophoreses and concentration was determined by UV absorbance. Affymetrix Arrays were performed with RNA samples from untreated and 6 h and 24 h TSA (50nM) and BMP2-treated cultures. RNA from cultures treated with TSA (10, 25, or 50nM) or BMP2 (10 ng/ml) from all three time points were used for quantitative real-time PCR.

### Biotin-labeled cDNA transcription and Affymetrix gene-chip hybridization

Total RNA samples obtained after 6 h and 24 h treatment were labeled and hybridized to an Affymetrix GeneChip® Mouse Genom 420 2.0 according to manufacturer’s protocol. Biotin-labled cRNA transcription and Affymetrix gene-chip hybridization was performed by the Genomic Core Facility of EMBL, Heidelberg.

### Analysis of gene expression data

Raw data obtained from Affymetrix gene-chip were analyzed using dChip (DNA-chip analyzer) software [31]. Samples were normalized using rank-based (quantile) normalization [52]. Genes were considered to be significantly regulated if expression had changed more than two-fold and absolute difference of normalized values exceeded 100 comparing treated and mock-treated samples with a confidence greater than 90%. Data was submitted to GEO (Gene Expression Omnibus) [GEO: GSE31792].

### Hierarchical clustering and functional annotation

In order to identify genes that respond similar to BMP2 and TSA treatment, we performed hierarchical clustering including probe sets regulated as described above (2-fold change, minimal euclidean distance 100) in any treatment group. Based on these criteria 2073 probe sets were included in the hierarchical clustering. The cluster analysis was done using dChip software [31]. Co-regulated genes identified in the cluster analysis were functionally annotated using DAVID (Database for Annotation, Visualization, and Integrated Discovery), a web based tool for functional annotation of genes according to the biological process they are involved in [32,33]. Additionally individual functional annotation clustering was performed with genes significantly regulated in one treatment group. In both cases genes were uploaded into DAVID using the web interface. Gene ontology (GO) terms were obtained including their p-value. GO terms with p-values < 10^-3^ were included in the further analysis.

### Reverse Transcription and real-time PCR

2 μg of total RNA extracted from neurosphere cultures was reverse transcribed using oligo(dT)_18_ primer (0.5 mg/ml, Fermentas) or random hexamer primers (100 μM, Fermentas) and SuperScript II reverse transcriptase (Invitrogen). Quantitative real-time PCR was performed on a LightCycler® 480 (Roche Applied Science) device using LightCycler® 480 SYBR Green I Master with 1 μl cDNA (1:5 dilution of transcribed cDNA). The following primer pairs were used:

Wisp1 5’: TGGACATCCAACTACACATCAA

Wisp1 3’: GGATGCAACACCTATTGTCAGT

Wnt5a 5’ TCAAGGACAGAAGAAACTCTGC

Wnt5a 3’: CACTGTGCTGCAGTTCCATCTC

Bambi 5’: ACGGACACCATTCCAAGAAG

Bambi 3’: CAGTGCACAAGGGAGAGGAT

Actb 5’: TTGCTGACAGGATGCAGAAG

Actb 3’: TGATCCACATCTGCTGGAAG

Gpr17 5’: CGACAGAAGAGCAAAGGGAC

Gpr17 3’: TCCTCTGACCCAAGTCTGCT

Id1 5’: CATGAACGGCTGCTACTCAC

Id1 3’: GTCCCGACTTCAGACTCCGAG

Id2 5’: GACTGCTACTCCAAGCTCAAG

Id2 3’: CACTATCGTCAGCCTGCATCAC.

The standard quantification protocol was applied with the following cycles: 1 cycle for preincubation: 5 min at 95°C, followed by 48 cycles for quantification: 10s at 95°C, 10s at 60°C 20s at 72°C. Melting curve analysis was performed for all samples in order to validate the unique generation of expected PCR products. In addition Stat3, Smad7, Bmp2 and Bmp4 expression was quantified using TaqMan assays (Applied Biosystems, Mm00456961_m1, Mm00484741_m1, Mm00432087_m1, Mm01340178_m1, Mm99999915_g1) Primer pairs recognizing beta-Actin or Gapdh were used for normalization.

For statistical analysis, relative expression (RE) levels were calculated with the function (RE = 2^-ΔΔCt^), where ΔΔCt is the normalized difference in threshold cycle (Ct) number between the control sample or the TSA- or BMP2-treated sample. Each Ct value was calculated from triplicate replicates of any given condition. The mean of relative expression levels were calculated from the individual RE values from 2–3 independent experiments, and the standard error of the mean (SEM) was calculated from the standard deviation. In order to evaluate the statistical significance the Student’s *T*-test was employed, comparing control sample to TSA- or BMP2-treated samples, respectively.

### Immunoblotting

Cells were washed once with room temperature PBS, then 200 μl lysis buffer (1 mM EDTA, 0.5% Triton-X-100, 6 M urea, in PBS, pH 7.2 - 7.4), complemented with 4% complete protein inhibitors (Roche), was added per plate. Cells were scraped from the plates on ice using cell scrapers (greiner bio-one). Lysates were transferred into eppendorf tubes, triturated through a syringe (0.80 x 40 mm 21 G, Braun Sterican) 10 times; the lysates were centrifuged at 13000 rpm for 12 min at 4°C, aliquoted and stored at −80°C. Protein concentration was determined via Bradford assay. Samples were then run on 15% SDS-gels, and blotted on PVDF-membranes (Millipore). For western blot analysis following primary antibodies were used: anti-pSmad1(Ser463/465)/5(Ser463/465)/8(Ser463/465) (Cell Signaling), anti-Smad1/5/8 (Santa Cruz), anti-pStat3(Tyr705) (Cell Signaling), anti-Stat3 (Cell Signaling), anti-pGsk3-beta(Ser9) (Cell Signaling), anti-Mbp (aa82-87) (AbD Serotec), anti-Gfap (DAKO), anti-Plp (aa3) (kind gift of Prof. J. Trotter, Mainz, Germany), and anti-beta-Actin (Sigma-Aldrich). As secondary antibody anti-mouse, anti-rat or anti-rabbit horseradish peroxidase (HRP)-conjugated antibodies (KPL) were used. Protein bands were visualized with Western Lightning ECL (Perkin Elmer) and detected with a luminescent image analyzer (LAS-3000, FujiFilm). For all western blots at least three repetitions were performed.

### ELISA Microarray

Phosphorylated proteins were quantified using an ArrayTube™ (Alere Technologies, Jena, Germany) based sandwich ELISA microarray, as previously described [52]. 10 μl of protein sample was applied on the microarray. Phosphorylated proteins were detected using commercially available isotype-specific capture antibodies and biotinylated phospho-specific detection antibodies (DuoSets IC kits, R&D Systems, Minneapolis, USA). For the detection the microarray was incubated with streptavidin–HRP conjugate (R&D Systems) followed by dye precipitation reaction using TrueBlue™ (KPL, Gaithersburg, MD, USA). Transmission was measured with the Arraymate™ reader (Alere Technologies) and protein concentration was quantified using standard calibration surfaces as described in Holenya et al. [53].

## Competing interest

The authors declare that they have no competing interests.

## Authors’ contributions

CS participated in the design of the study, carried out gene expression analysis, performed qRT-PCR and Western Blot and drafted the manuscript. KW participated in the design of the study, carried out tissue culture, performed immunocytofluorescent stainings, qRT-PCR, and Western Blot and drafted the manuscript. PH carried out the ELISA microarray. MSR participated in the tissue culture and design of gene expression profiling. KLT participated in the design and coordination of the study, carried out confocal analysis, and drafted the manuscript. SW participated in the design and coordination of the study, carried out gene expression analysis and drafted the manuscript. All authors read and approved the final manuscript.

## Supplementary Material

Additional file 1 **Table S1 - S26.** Additional file 1 contains tables with additional gene expression data. Table S1-S4 summarize the 25 genes with the strongest regulation of expression after TSA or BMP2 treatment. Table S5-S10 contains the genes regulated in two treatments, which represents the intersections of the Venn Diagrams from Figure 3A-F. Table S11-S21 contains the GO terms obtained from the of hierarchical cluster analysis (Figure 3G). Table S22-S26 contains the GO terms from functional annotation cluster (Figure 4A-E) of all genes regulated within an individual treatment. Click here for file
