# Supplementary material for: Distinct and overlapping gene regulatory networks in BMP- and HDAC-controlled cell fate determination in the embryonic forebrain
Source: BMC Genomics. 2012 Jul 2;13:298. doi: 10.1186/1471-2164-13-298 (PMC3460768; doi:10.1186/1471-2164-13-298)
Supplement: Additional file 1 — Table S1 - S26. Additional file 1 contains tables with additional gene expression data. Table S1-S4 summarize the 25 genes with the strongest regulation of expression after TSA or BMP2 treatment. Table S5-S10 contains the genes regulated in two treatments, which represents the intersections of the Venn Diagrams from Figure 3A-F. Table S11-S21 contains the GO terms obtained from the of hierarchical cluster analysis (Figure 3G). Table S22-S26 contains the GO terms from functional annotation cluster (Figure 4A-E) of all genes regulated within an individual treatment. [file 1471-2164-13-298-S1.pdf]

Supplementary Data:

Table S1:
25 Genes with the strongest Regulation of Expression after Treatment with **BMP2 6h**

top regulated Genes	Gene Symbol	Fold Change	Difference of Means
**Induced**			
Wnt inhibitory factor 1	Wif1	107.77	397.32
connective tissue growth factor	Ctgf	32.22	728.34
MAD homolog 6 (Drosophila)	Smad6	26.68	585.58
gap junction protein, alpha 3	Gja3	26.18	112.25
noggin	Nog	25.14	2961.24
myocilin	Myoc	20.14	118.14
atonal homolog 8 (Drosophila)	Atoh8	17.18	283.24
follistatin	Fst	16.56	580.48
ADAMTS-like 2	Adamtsl2	15.45	168.23
Mm,103566,1		12.3	103.04
DIRAS family, GTP-binding RAS-like 2	Diras2	12.12	350.37
RIKEN cDNA A730054J21 gene	A730054J21Rik	10.66	424.79
kinesin family member 26B	Kif26b	10.6	308.18
growth arrest and DNA-damage-inducible 45 beta	Gadd45b	9.84	246.59
cyclin-dependent kinase inhibitor 1C (P57)	Cdkn1c	9.28	1572.64
follistatin	Fst	9.2	132.86
tumor necrosis factor receptor superfamily, member 12a	Tnfrsf12a	9.04	254.32
3'-phosphoadenosine 5'-phosphosulfate synthase 2	Papss2	8.87	393.89
Mm,197257,1		8.6	186.26
**Supressed**			
3'-phosphoadenosine 5'-phosphosulfate synthase 2	Papss2	7.77	555.95
bone morphogenetic protein 4	Bmp4	-9.01	-865.31
protein tyrosine phosphatase, receptor type, E	Ptpre	-11.36	-308.04
expressed sequence AI414108	AI414108	-14.08	-338.03
chondroitin sulfate proteoglycan 4	Cspg4	-14.6	-159.76
protein tyrosine phosphatase, receptor type, E	Ptpre	-18.37	-762.52

Table S2:
25 Genes with the strongest Regulation of Expression after Treatment with **BMP2 24h**

top regulated Genes	Gene Symbol	Fold Change	Difference of Means
**Induced**			
Wnt inhibitory factor 1	Wif1	273.64	1325.14
gap junction protein, alpha 3	Gja3	255.35	738.33
Mm,103566,1		101.11	633.16
DIRAS family, GTP-binding RAS-like 2	Diras2	99.94	3457.18
tumor necrosis factor receptor superfamily, member 13c	Tnfrsf13c	98.72	369.56
neuropeptide VF precursor	Npvf	85.65	847.12
serine (or cysteine) peptidase inhibitor, clade F, member 1	Serpinf1	83.55	1704.52
lysozyme 1	Lyz1	82.55	202.57
ADAMTS-like 2	Adamtsl2	79.26	517.68
predicted gene 6041	Gm6041	67.14	300.86
serine (or cysteine) peptidase inhibitor, clade F, member 1	Serpinf1	50.46	829.96
fibromodulin	Fmod	44.97	357.44
cell death-inducing DNA fragmentation factor, alpha subunit-like effector A	Cidea	40.68	922.63
apelin receptor	Aplnr	40.55	246.76
RIKEN cDNA A730054J21 gene	A730054J21Rik	37.09	939.5
expressed sequence C86753	C86753	37.09	274.31
family with sequence similarity 101, member A	Fam101a	36.03	276.81
lysozyme 1	Lyz1	35.27	114.69
gastrulation brain homeobox 2	Gbx2	33.79	141.95
predicted gene 10001	Gm10001	32.41	1265.2
secretogranin II	Scg2	29.99	871.22
MAD homolog 6 (Drosophila)	Smad6	29.71	807.36
predicted gene 14134	Gm14134	29.55	248.19
**Supressed**			
G protein-coupled receptor 17	Gpr17	-30.9	-3832.45
bone morphogenetic protein 4	Bmp4	-34.17	-1166.86

Table S3:
25 Genes with the strongest Regulation of Expression after Treatment with **TSA 6h**

top regulated Genes	Gene Symbol	Fold Change	Difference of Means
**Induced**			
Ras association (RalGDS/AF-6) domain family member 4	Rassf4	28.23	188.97
Mm,40442,1	Efna3	26.26	316.59
Histone cluster 1, H1e	Hist1h1e	25.22	1183.64
immediate early response 3	Ier3	23.54	171.57
Histone cluster 1, H1e	Hist1h1e	21.38	422.51
interferon zeta	Ifnz	17.79	242.82
ephrin A3 /// similar to Ephrin A3	Efna3 /// LOC100046031	17.24	734.87
FXYD domain-containing ion transport regulator 7	Fxyd7	17.14	125.99
transcription factor-like 5 (basic helix-loop-helix)	Tcfl5	16.43	325.15
testis expressed gene 15	Tex15	16.33	158.68
similar to Tescalcin /// tescalcin	LOC100047138 /// Tesc	13.81	301.85
protease, serine, 16 (thymus)	Prss16	13.1	118.15
desert hedgehog	Dhh	12.84	167.56
insulin-like 6	Insl6	12.82	184.99
receptor accessory protein 6	Reep6	12.23	428.33
heat shock protein 1B	Hspa1b	12.11	760.82
similar to Y box protein 2 /// Y box protein 2	LOC100045903 /// Ybx2	11.99	130.57
heat shock protein 1A	Hspa1a	11.83	400.14
**Supressed**			
protein kinase C, theta	Prkcq	-11.82	-820.17
adenosine monophosphate deaminase 3	Ampd3	-12.29	-393.95
RIKEN cDNA 9630013A20 gene	9630013A20Rik	-12.52	-936.73
RIKEN cDNA A230069A22 gene	A230069A22Rik	-14.4	-723.81
breast carcinoma amplified sequence 1	Bcas1	-14.56	-327.11
H2A histone family, member Y2	H2afy2	-14.59	-536.91
distal-less homeobox 1, antisense	Dlx1as	-19.97	-209.32

Table S4:
25 Genes with the strongest Regulation of Expression after Treatment with **TSA 24h**

top regulated Genes	Gene Symbol	Fold Change	Difference of Means
**Induced**			
heat shock protein 1	Hspb1	155.85	169.78
histocompatibility 2, class II antigen A, beta 1	H2-Ab1	135.41	208.61
ribonuclease, RNase A family 4	Rnase4	117.69	646.19
histocompatibility 2, class II antigen A, beta 1	H2-Ab1	106.4	475.16
uncoupling protein 2 (mitochondrial, proton carrier)	Ucp2	100.18	245.58
RAB26, member RAS oncogene family	Rab26	64.17	153.5
FXYD domain-containing ion transport regulator 7	Fxyd7	52.15	335.33
RIKEN cDNA A730017C20 gene	A730017C20 Rik	50.87	385.84
RIKEN cDNA C920025E04 gene	C920025E04Rik	49.53	326.55
glutathione S-transferase, theta 3	Gstt3	47.56	205.74
**Supressed**			
ermin, ERM-like protein	Ermn	-47.23	-761.25
hypothetical protein LOC100047091 /// transmembrane protein 163	LOC100047091 /// Tmem163	-51.24	-924.17
myelin-associated glycoprotein	Mag	-57.29	-3581.1
gap junction protein, gamma 2	Gjc2	-69.07	-379.46
sphingosine-1-phosphate receptor 5	S1pr5	-75.01	-197.56
transmembrane protein 125	Tmem125	-77.81	-386.8
expressed sequence AI314604	AI314604	-78.81	-2677.05
G protein-coupled receptor 17	Gpr17	-80.21	-4729.8
myelin-associated oligodendrocytic basic protein	Mobp	-105.64	-3013.77
RIKEN cDNA 9630013A20 gene	9630013A20Rik	-122.52	-1083.54
myelin-associated oligodendrocytic basic protein	Mobp	-124.67	-2332.93
myelin-associated oligodendrocytic basic protein	Mobp	-132.05	-3959.17
RIKEN cDNA 9630013A20 gene	9630013A20Rik	-135.9	-1530.53
myelin oligodendrocyte glycoprotein	Mog	-148.66	-501.63
RIKEN cDNA 9630013A20 gene	9630013A20Rik	-169.41	-909

Table S5:
Significant regulated Genes after **BMP2 6h and 24h**. (Intersection Fig. 3E)
ESTs and non-annotated Sequences are excluded.

Regulated Genes BMP2 6h and 24h	Symbol	Fold Change BMP2 6h	Fold Change BMP2 24h
Wnt inhibitory factor 1	Wif1	107.77	273.64
MAD homolog 6 (Drosophila)	Smad6	26.68	29.71
gap junction protein, alpha 3	Gja3	26.18	255.35
noggin	Nog	25.14	16.22
myocilin	Myoc	20.14	23.7
atonal homolog 8 (Drosophila)	Atoh8	17.18	20.87
follistatin	Fst	16.56	19.63
ADAMTS-like 2	Adamtsl2	15.45	79.26
DIRAS family, GTP-binding RAS-like 2	Diras2	12.12	99.94
kinesin family member 26B	Kif26b	10.6	22.1
growth arrest and DNA-damage-inducible 45 beta	Gadd45b	9.84	14.4
tumor necrosis factor receptor superfamily, member 12a	Tnfrsf12a	9.04	10.1
3'-phosphoadenosine 5'-phosphosulfate synthase 2	Papss2	7.77	16.72
myosin regulatory light chain interacting protein	Mylip	7.66	4.1
fos-like antigen 2 /// similar to fos-like antigen 2	Fosl2 /// LOC634417	7.48	6.19
zinc finger and BTB domain containing 7C	Zbtb7c	7.28	6.5
family with sequence similarity 70, member A	Fam70a	7.28	13.35
serine/threonine kinase 32B	Stk32b	7.26	25.15
immediate early response 5-like	Ier5l	6.71	5.79
naked cuticle 1 homolog (Drosophila)	Nkd1	6.35	4.7
cadherin 22 /// similar to cadherin 22	Cdh22 /// LOC100046008	6.25	8.83
serine (or cysteine) peptidase inhibitor, clade F, member 1	Serpinf1	5.95	83.55
patched domain containing 2	Ptchd2	5.77	7.31
transmembrane protein 158	Tmem158	5.68	8.71
predicted gene 10001	Gm10001	5.32	32.41
NUAK family, SNF1-like kinase, 1	Nuak1	5.31	10.81
regulator of G-protein signaling 2	Rgs2	5.2	5.45
Src homology 2 domain containing F	Shf	5.07	4.65
solute carrier family 2 (facilitated glucose transporter), member 13	Slc2a13	5.05	4.81
MAD homolog 7 (Drosophila)	Smad7	4.96	6.5
protein tyrosine phosphatase, receptor type, U	Ptpru	4.86	11.55
glutamate receptor ionotropic, NMDA3A	Grin3a	4.85	4.55
HOP homeobox	Hopx	4.76	19.68
mannoside acetylglucosaminyltransferase 4, isoenzyme A	Mgat4a	4.64	5.47
potassium voltage gated channel, Shaw-related subfamily, member 4	Kcnc4	4.59	7.72
Rhesus blood group-associated C glycoprotein	Rhcg	4.37	5.5
Ly6/Plaur domain containing 1	Lypd1	4.34	6.07
hairy/enhancer-of-split related with YRPW motif 1	Hey1	4.18	3.55
adenylate cyclase 1	Adcy1	4.13	6.33

continue

Table S5 (Part 2)

Regulated Genes BMP2 6h and 24h	Symbol	Fold Change BMP2 6h	Fold Change BMP2 24h
cannabinoid receptor 1 (brain)	Cnr1	3.99	5.56
collagen, type V, alpha 3	Col5a3	3.94	8.35
family with sequence similarity 46, member A	Fam46a	3.84	10.67
WW, C2 and coiled-coil domain containing 2	Wwc2	3.71	3.67
potassium channel, subfamily K, member 13	Kcnk13	3.64	7.58
cartilage acidic protein 1	Crtac1	3.47	3.56
BMP and activin membrane-bound inhibitor, homolog (Xenopus laevis)	Bambi	3.46	3.6
vasorin	Vasn	3.45	5.06
CXXC finger 5	Cxxc5	3.34	3.76
transmembrane protein 64	Tmem64	3.28	2.91
Rho guanine nucleotide exchange factor (GEF) 4	Arhgef4	3.11	2.54
inhibitor of DNA binding 1	Id1	3.08	2.2
zinc finger protein 423	Zfp423	3.06	3.61
olfactomedin 2	Olfm2	2.97	5.14
cut-like homeobox 2	Cux2	2.93	4.1
Ca2+-dependent secretion activator	Cadps	2.9	4.96
nuclear factor of kappa light polypeptide gene enhancer in B-cells inhibitor, alpha	Nfkbia	2.77	2.89
PDZ domain containing RING finger 3	Pdzrn3	2.74	3.31
neuropilin 1	Nrp1	2.72	2.77
ninjurin 1	Ninj1	2.7	6.31
ribosomal protein S6 kinase, polypeptide 5	Rps6ka5	2.5	6.24
family with sequence similarity 129, member B	Fam129b	2.43	3.32
bone morphogenetic protein receptor, type 1A	Bmpr1a	2.31	2.52
GH regulated TBC protein 1	Grtp1	-2.31	-3.06
myelocytomatosis oncogene	Myc	-2.37	-4.17
erythrocyte protein band 4,1	Epb4,1	-2.43	-3.67
Ras association (RalGDS/AF-6) domain family member 2	Rassf2	-2.45	-3.26
carbonyl reductase 3	Cbr3	-2.46	-3.28
leucine rich repeat protein 1, neuronal	Lrrn1	-2.52	-2.82
GS homeobox 1	Gsx1	-2.52	-5.17
EF hand domain containing 2	Efhd2	-2.57	-4.53
carbonic anhydrase 8	Car8	-2.65	-6.46
LIM and senescent cell antigen like domains 2	Lims2	-2.8	-24.72
breast carcinoma amplified sequence 1	Bcas1	-2.8	-24.38
LIM domain and actin binding 1	Lima1	-2.86	-3.5
glycerol-3-phosphate dehydrogenase 1 (soluble)	Gpd1	-2.93	-8.02
methylthioadenosine phosphorylase	Mtap	-3.08	-3.27
inhibin beta-B /// similar to Inhbb protein	Inhbb /// LOC100046802	-3.1	-4.22
G protein-coupled receptor 17	Gpr17	-3.13	-30.9
SPARC related modular calcium binding 1	Smoc1	-3.15	-3.95
phosphatase and actin regulator 3	Phactr3	-3.18	-6.73

continue

Table S5 (Part 3)

Regulated Genes BMP2 6h and 24h	Symbol	Fold Change BMP2 6h	Fold Change BMP2 24h
SH3-domain binding protein 4	Sh3bp4	-3.26	-4.35
seizure related 6homolog like	Sez6l	-3.3	-2.91
phospholipase C, gamma 2	Plcg2	-3.39	-9.29
actin filament associated protein 1-like 2	Afap1l2	-3.47	-3.68
TNF receptor associated factor 4	Traf4	-3.54	-4.89
B-cell translocation gene 2, anti-proliferative	Btg2	-3.64	-3.71
zinc finger protein 365	Zfp365	-3.75	-5.3
GS homeobox 2	Gsx2	-3.85	-8.02
G0/G1 switch gene 2	G0s2	-3.89	-5.35
similar to manic fringe /// MFNG O-fucosylpeptide 3-beta-N-acetylglucosaminyltransferase	LOC100046464 /// Mfng	-3.94	-4.47
neurocalcin delta	Ncald	-3.94	-3.68
leucine rich repeat and fibronectin type III, extracellular 1	Elfn1	-4.08	-6.57
delta-like 3 (Drosophila)	Dll3	-4.14	-3.64
epidermal growth factor receptor	Egfr	-4.15	-7.93
delta-like 1 (Drosophila)	Dll1	-4.59	-4.19
nuclear factor, erythroid derived 2, like 3	Nfe2l3	-4.66	-26.54
transmembrane protein 51	Tmem51	-4.83	-4.3
pleckstrin homology domain containing, family F (with FYVE domain) member 1	Plekhf1	-5.88	-3.04
amiloride-sensitive cation channel 2, neuronal	Accn2	-6.07	-9.61
bone morphogenetic protein 4	Bmp4	-9.01	-34.17
protein tyrosine phosphatase, receptor type, E	Ptpre	-11.36	-11.71
chondroitin sulfate proteoglycan 4	Cspg4	-14.6	-4.05

Table S6:
Significant regulated Genes after **BMP2 6h and TSA 6h**. (Intersection Fig. 3A)
ESTs and non-annotated Sequences are excluded.

**Regulated Genes BMP2 6h and TSA 6h**	**Symbol**	**Fold Change BMP2 6h**	**Fold Change TSA 6h**
tumor necrosis factor receptor superfamily, member 12a	Tnfrsf12a	9.04	7.39
myosin regulatory light chain interacting protein	Mylip	7.66	3.47
family with sequence similarity 19, member A2	Fam19a2	3.13	-3.1
Ca2+-dependent secretion activator	Cadps	2.9	-2.52
serum/glucocorticoid regulated kinase 3	Sgk3	2.65	-2.54
Six3 opposite strand transcript 1	Six3os1	2.51	-4.66
solute carrier organic anion transporter family, member 1c1	Slco1c1	-2.2	-2.42
myelocytomatosis oncogene	Myc	-2.37	-3.82
solute carrier family 24 (sodium/potassium/calcium exchanger), member 3	Slc24a3	-2.44	-3.79
GS homeobox 1	Gsx1	-2.52	-4.86
carbonic anhydrase 8	Car8	-2.65	2.65
LIM and senescent cell antigen like domains 2	Lims2	-2.8	-4.61
breast carcinoma amplified sequence 1	Bcas1	-2.8	-9.53
BTB (POZ) domain containing 17	Btbd17	-2.82	-8.1
glycerol-3-phosphate dehydrogenase 1 (soluble)	Gpd1	-2.93	-2.5
G protein-coupled receptor 17	Gpr17	-3.13	-6.8
Down syndrome cell adhesion molecule-like 1	Dscaml1	-3.25	-3.19
seizure related 6homolog like	Sez6l	-3.3	-2.92
actin filament associated protein 1-like 2	Afap1l2	-3.47	-4.84
G0/G1 switch gene 2	G0s2	-3.89	-6.74
leucine rich repeat and fibronectin type III, extracellular 1	Elfn1	-4.08	-2.81
delta-like 3 (Drosophila)	Dll3	-4.14	-3.32
protein tyrosine phosphatase, receptor type, E	Ptpre	-18.37	-2.53

Table S7:
Significant regulated Genes after **BMP2 6h and TSA 24h**. (Intersection Fig. 3B)
ESTs and non-annotated Sequences are excluded.

**Regulated Genes BMP2 6h and TSA 24h**	**Symbol**	**Fold Change BMP2 6h**	**Fold Change TSA 24h**
connective tissue growth factor	Ctgf	32.22	5.94
follistatin	Fst	16.56	4.18
DIRAS family, GTP-binding RAS-like 2	Diras2	12.12	4.51
family with sequence similarity 70, member A	Fam70a	7.28	16.08
3'-phosphoadenosine 5'-phosphosulfate synthase 2	Papss2	6.9	-3.34
MAD homolog 7 (Drosophila)	Smad7	4.96	-5.03
ring finger protein 128	Rnf128	4.55	5.47
BMP and activin membrane-bound inhibitor, homolog (Xenopus laevis)	Bambi	3.46	3.41
family with sequence similarity 19, member A2	Fam19a2	3.13	-5.38
calcium channel, voltage-dependent, alpha 2/delta subunit 2	Cacna2d2	2.65	3.81
lysyl oxidase-like 1	Loxl1	2.39	3.56
zinc finger, DHHC domain containing 14	Zdhhc14	-2.25	-3.75
erythrocyte protein band 4,1	Epb4,1	-2.43	-2.55
solute carrier family 24 (sodium/potassium/calcium exchanger), member 3	Slc24a3	-2.44	-4.96
LIM and senescent cell antigen like domains 2	Lims2	-2.8	-27.27
breast carcinoma amplified sequence 1	Bcas1	-2.8	-17.36
glycerol-3-phosphate dehydrogenase 1 (soluble)	Gpd1	-2.93	-7.87
G protein-coupled receptor 17	Gpr17	-3.13	-80.21
phosphatase and actin regulator 3	Phactr3	-3.18	-5.81
Down syndrome cell adhesion molecule-like 1	Dscaml1	-3.25	-5.91
SH3-domain binding protein 4	Sh3bp4	-3.26	-2.79
actin filament associated protein 1-like 2	Afap1l2	-3.47	-4.16
TNF receptor associated factor 4	Traf4	-3.54	-2.45
zinc finger protein 365	Zfp365	-3.75	-3.23
G0/G1 switch gene 2	G0s2	-3.89	-4.02
delta-like 3 (Drosophila)	Dll3	-4.14	-4.49
epidermal growth factor receptor	Egfr	-4.15	2.73
bone morphogenetic protein 4	Bmp4	-9.01	-15.47
protein tyrosine phosphatase, receptor type, E	Ptpre	-11.36	-5.93

Table S8:
Significant regulated Genes after **BMP2 24h and TSA 6h**. (Intersection Fig. 3C)
ESTs and non-annotated Sequences are excluded.

Regulated Genes BMP2 24h and TSA 6h	Symbol	Fold Change BMP2 24h	Fold Change TSA 6h
Kruppel-like factor 4 (gut)	Klf4	13.51	7.94
tubulin, beta 6	Tubb6	12.04	7.41
tumor necrosis factor receptor superfamily, member 12a	Tnfrsf12a	10.1	7.39
Rho GTPase activating protein 29	Arhgap29	8.01	5.84
KDEL (Lys-Asp-Glu-Leu) endoplasmic reticulum protein retention receptor 3	Kdelr3	7.32	11.15
aquaporin 11	Aqp11	5.08	3.24
Ca2+-dependent secretion activator	Cadps	4.96	-2.52
zinc finger, CCHC domain containing 12	Zcchc12	4.24	8.5
myosin regulatory light chain interacting protein	Mylip	4.1	3.47
growth factor receptor bound protein 14	Grb14	3.43	2.87
regulator of G-protein signalling 10	Rgs10	3.25	3.2
bone morphogenetic protein receptor, type 1A	Bmpr1a	2.47	-2.23
striatin, calmodulin binding protein	Strn	-2.35	-2.94
oligodendrocyte myelin glycoprotein	Omg	-2.61	-11.48
coatomer protein complex, subunit gamma 2, antisense 2	Copg2as2	-2.64	2.41
WAS/WASL interacting protein family, member 1	Wipf1	-2.68	-3.89
ring finger protein 122	Rnf122	-2.76	-2.63
adenosine monophosphate deaminase 3	Ampd3	-2.77	-12.29
shroom family member 2	Shroom2	-2.78	-3.69
zinc fingerprotein 618	Zfp618	-2.84	-4.19
seizure related 6homolog like	Sez6l	-2.91	-2.92
dysbindin (dystrobrevin binding protein 1) domain containing 2	Dbndd2	-2.92	-3.18
erythrocyte protein band 4,1-like 3	Epb4,1l3	-3.1	-2.41
CUB and Sushi multiple domains 2	Csmd2	-3.14	-3.08
proline rich region 18	Prr18	-3.19	-8.52
SRY-box containing gene 10	Sox10	-3.21	-3.18
S100 protein, beta polypeptide, neural	S100b	-3.21	-3.78
chimerin (chimaerin) 2	Chn2	-3.44	-5.09
delta-like 3 (Drosophila)	Dll3	-3.64	-3.32
actin filament associated protein 1-like 2	Afap1l2	-3.68	-4.84
family with sequence similarity 13, member C	Fam13c	-4.16	-4.83
myelocytomatosis oncogene	Myc	-4.17	-3.82
cytoplasmic FMR1 interacting protein 2	Cyfip2	-4.22	-2.53
leucine rich repeat transmembrane neuronal 1	Lrrtm1	-4.49	-7.54
protein kinase C, theta	Prkcq	-4.7	-11.82
Down syndrome cell adhesion molecule	Dscam	-4.85	-3.7
G protein-coupled receptor 149	Gpr149	-4.87	-6.66
platelet derived growth factor, alpha	Pdgfa	-5.08	-4.83
GS homeobox 1	Gsx1	-5.17	-4.86
G0/G1 switch gene 2	G0s2	-5.35	-6.74

continue

Table S8 (Part 2)

**Regulated Genes BMP2 24h and TSA 6h**	**Symbol**	**Fold Change BMP2 24h**	**Fold Change TSA 6h**
EF hand domain containing 1	Efhd1	-5.45	3.22
dedicator of cytokinesis 9	Dock9	-5.59	-2.63
ELOVL family member 7, elongation of long chain fatty acids (yeast)	Elovl7	-5.71	-3.33
G protein-coupled receptor 37-like 1	Gpr37l1	-5.74	-4.21
tubulin, beta 4	Tubb4	-5.93	-4.74
carbonic anhydrase 8	Car8	-6.46	2.65
leucine rich repeat and fibronectin type III, extracellular 1	Elfn1	-6.57	-2.81
plexin B3	Plxnb3	-6.81	-4.09
serine/arginine-rich protein specific kinase 3	Srpk3	-7.54	-6.01
Ras association (RalGDS/AF-6) domain family (N-terminal) member 10	Rassf10	-7.88	-3.37
glycerol-3-phosphate dehydrogenase 1 (soluble)	Gpd1	-8.02	-2.5
platelet derived growth factor receptor, alpha polypeptide	Pdgfra	-8.15	-3.93
galactose-3-O-sulfotransferase 1	Gal3st1	-8.92	-4.96
RAB33A, member of RAS oncogene family	Rab33a	-9.41	-7.1
breast carcinoma amplified sequence 1	Bcas1	-11.02	-2.93
four and a half LIM domains 2	Fhl2	-11.15	-6.79
predicted gene 98	Gm98	-13.78	-4.13
ectonucleotide pyrophosphatase/phosphodiesterase 6	Enpp6	-16.26	-2.98
protein tyrosine phosphatase, receptor type, E	Ptpre	-16.45	-2.53
LIM and senescent cell antigen like domains 2	Lims2	-24.72	-4.61
G protein-coupled receptor 17	Gpr17	-30.9	-6.8

Table S9:
Significant regulated Genes after **BMP2 24h and TSA 24h**. (Intersection Fig. 3D)
ESTs and non-annotated Sequences are excluded.

**Regulated Genes BMP2 24h and TSA 24h**	**Symbol**	**Fold Change BMP2 24h**	**Fold Change TSA 24h**
DIRAS family, GTP-binding RAS-like 2	Diras2	99.94	4.51
family with sequence similarity 101, member A	Fam101a	36.03	16.73
follistatin	Fst	19.63	4.18
aldehyde dehydrogenase family 1, subfamily A7	Aldh1a7	17.98	7.23
Solute carrier family 26, member 7	Slc26a7	15.08	6.15
Purkinje cell protein 4	Pcp4	14.04	8.63
Kruppel-like factor 4 (gut)	Klf4	13.51	4.74
family with sequence similarity 70, member A	Fam70a	13.35	16.08
3'-phosphoadenosine 5'-phosphosulfate synthase 2	Papss2	10.7	-3.34
selenoprotein M	Selm	9.08	4.39
sema domain, immunoglobulin domain (Ig), short basic domain, secreted, (semaphorin) 3C	Sema3c	8.52	4.87
Rho GTPase activating protein 29	Arhgap29	8.01	4.35
protocadherin 20	Pcdh20	7.91	17.1
KDEL (Lys-Asp-Glu-Leu) endoplasmic reticulum protein retention receptor 3	Kdelr3	7.32	6.87
nephronectin	Npnt	7.21	5.04
MAD homolog 7 (Drosophila)	Smad7	6.86	-5.69
expressed sequence AW551984	AW551984	6.77	12.53
guanylate cyclase 1, soluble, alpha 3	Gucy1a3	6.21	5.18
phosphodiesterase 8A	Pde8a	5.36	4.27
aquaporin 11	Aqp11	5.08	4.61
tumor protein D52-like 1	Tpd52l1	4.68	7.97
calsyntenin 2	Clstn2	4.68	4.95
retinoic acid receptor responder (tazarotene induced) 1	Rarres1	4.45	2.96
zinc finger, CCHC domain containing 12	Zcchc12	4.24	9.35
fibronectin 1	Fn1	4.12	4.78
eyes absent 4 homolog (Drosophila)	Eya4	4.07	3.09
interferon induced transmembrane protein 2	Ifitm2	3.91	10.33
a disintegrin and metallopeptidase domain 23	Adam23	3.84	3.13
BMP and activin membrane-bound inhibitor, homolog (Xenopus laevis)	Bambi	3.6	3.41
growth arrest specific 6	Gas6	3.49	3.71
BCL2-associated athanogene 3	Bag3	3.44	3.06
neuron specific gene family member 2	Nsg2	3.4	2.65
Eph receptor A5	Epha5	3.26	2.57
regulator of G-protein signalling 10	Rgs10	3.25	6.39
insulin-like growth factor binding protein 3	Igfbp3	2.76	3.06
TBC1 domain family, member 8B	Tbc1d8b	2.43	2.9
striatin, calmodulin binding protein	Strn	-2.35	-4.07
tyrosine kinase, non-receptor, 2	Tnk2	-2.37	-2.71
p21 protein (Cdc42/Rac)-activated kinase 3	Pak3	-2.47	-2.69
oligodendrocyte myelin glycoprotein	Omg	-2.61	-9.86

continue

Table S9 (Part 2)

Regulated Genes BMP2 24h and TSA 24h	Symbol	Fold Change BMP2 24h	Fold Change TSA 24h
Cnksr family member 3	Cnksr3	-2.62	-3.73
WAS/WASL interacting protein family, member 1	Wipf1	-2.68	-4.2
phosphodiesterase 9A	Pde9a	-2.68	-2.23
ring finger protein 122	Rnf122	-2.76	-2.92
pellino 1	Peli1	-2.77	-4.66
shroom family member 2	Shroom2	-2.78	-4.46
dedicator of cytokinesis 10	Dock10	-2.78	-2.63
bridging integrator 1	Bin1	-2.83	-3.8
erythrocyte protein band 4,1-like 3	Epb4,1l3	-2.87	-3.35
glycolipid transfer protein	Gltp	-2.88	-2.82
transforming growth factor alpha	Tgfa	-2.89	-3.99
sirtuin 2 (silent mating type information regulation 2, homolog) 2 (S, cerevisiae)	Sirt2	-2.97	-2.9
pleckstrin homology-like domain, family B, member 1	Phldb1	-3.04	-3.94
myelin basic protein	Mbp	-3.11	-8.3
proline rich region 18	Prr18	-3.19	-4.58
SRY-box containing gene 10	Sox10	-3.21	-5.31
S100 protein, beta polypeptide, neural	S100b	-3.21	-11.6
cytoplasmic FMR1 interacting protein 2	Cyfip2	-3.24	-2.66
chimerin (chimaerin) 2	Chn2	-3.44	-7.68
protein kinase C, zeta	Prkcz	-3.48	-6.68
similar to voltage gated channel like 1 /// sodium leak channel, non-selective	LOC675405 /// Nalcn	-3.63	-8.52
SH3-domain GRB2-like 3	Sh3gl3	-3.64	-7.51
delta-like 3 (Drosophila)	Dll3	-3.64	-4.49
erythrocyte protein band 4,1	Epb4,1	-3.67	-2.55
actin filament associated protein 1-like 2	Afap1l2	-3.68	-4.16
proteolipid protein (myelin) 1	Plp1	-3.72	-8.14
ELOVL family member 7, elongation of long chain fatty acids (yeast)	Elovl7	-3.82	-6.76
lysophosphatidylglycerol acyltransferase 1	Lpgat1	-3.89	-5.79
dynamin 3	Dnm3	-3.9	-6.71
neurofascin	Nfasc	-3.98	-8.21
dysbindin (dystrobrevin binding protein 1) domain containing 2	Dbndd2	-3.99	-2.77
2',3'-cyclic nucleotide 3' phosphodiesterase	Cnp	-4.05	-5.01
parvin, beta	Parvb	-4.1	-3.1
ankyrin 3, epithelial	Ank3	-4.22	-5.79
SH3-domain binding protein 4	Sh3bp4	-4.26	-3.11
dedicator of cytokinesis 9	Dock9	-4.3	-3.05
leucine rich repeat transmembrane neuronal 1	Lrrtm1	-4.49	-5.2
UDP galactosyltransferase 8A	Ugt8a	-4.53	-10.14
protein kinase C, theta	Prkcq	-4.7	-12.45
brain and acute leukemia, cytoplasmic	Baalc	-4.71	-3.69
Down syndrome cell adhesion molecule	Dscam	-4.85	-11.67

continue

Table S9 (Part 3)

Regulated Genes BMP2 24h and TSA 24h	Symbol	Fold Change BMP2 24h	Fold Change TSA 24h
TNF receptor associated factor 4	Traf4	-4.89	-2.45
sorting nexin family member 30	Snx30	-5	-4.86
fatty acid 2-hydroxylase	Fa2h	-5.13	-11.66
family with sequence similarity 5, member C	Fam5c	-5.28	-4.41
zinc finger protein 365	Zfp365	-5.3	-3.23
G0/G1 switch gene 2	G0s2	-5.35	-4.02
EF hand domain containing 1	Efhd1	-5.45	6.77
platelet derived growth factor, alpha	Pdgfa	-5.57	-3.59
tubulin, beta 4	Tubb4	-5.93	-12.31
phosphatase and actin regulator 3	Phactr3	-6.73	-5.81
plexin B3	Plxnb3	-6.81	-19.44
monooxygenase, DBH-like 1	Moxd1	-6.85	5.89
serine/arginine-rich protein specific kinase 3	Srpk3	-7.54	-26.6
protein phosphatase 1, regulatory (inhibitor) subunit 16B	Ppp1r16b	-7.6	-10.53
dual specificity phosphatase-like 15	Dusp15	-7.73	-3.65
epidermal growth factor receptor	Egfr	-7.93	2.73
glycerol-3-phosphate dehydrogenase 1 (soluble)	Gpd1	-8.02	-7.87
inositol 1,4,5-triphosphate receptor 2	Itpr2	-8.23	-4.25
immunoglobulin-like domain containing receptor 2	Ildr2	-8.28	8.47
galactose-3-O-sulfotransferase 1	Gal3st1	-8.92	-18.2
RAB33A, member of RAS oncogene family	Rab33a	-9.41	-13.66
dipeptidylpeptidase 10 /// similar to Dipeptidylpeptidase 10	Dpp10 /// LOC100047231	-10.14	-4
breast carcinoma amplified sequence 1	Bcas1	-11.02	-13.89
four and a half LIM domains 2	Fhl2	-11.15	-9.48
protein tyrosine phosphatase, receptor type, E	Ptpre	-11.71	-5.93
cerebellin 2 precursor protein	Cbln2	-12.09	-11.23
oculocutaneous albinism II	Oca2	-14.15	-9.37
ectonucleotide pyrophosphatase/phosphodiesterase 6	Enpp6	-16.26	-33.26
protocadherin 15	Pcdh15	-17.37	-6.63
LIM and senescent cell antigen like domains 2	Lims2	-24.72	-27.27
G protein-coupled receptor 17	Gpr17	-30.9	-80.21
bone morphogenetic protein 4	Bmp4	-34.17	-15.47

Table S10:
Significant regulated Genes after **TSA 6h and TSA 24h**. (Intersection Fig. 3F)
ESTs and non-annotated Sequences are excluded.

Regulated Genes TSA 6h and TSA 24h	Symbol	Fold Change TSA 6h	Fold Change TSA 24h
Ras association (RalGDS/AF-6) domain family member 4	Rassf4	28.23	17.33
immediate early response 3	Ier3	23.54	10.77
interferon zeta	Ifnz	17.79	7.17
ephrin A3 /// similar to Ephrin A3	Efna3 /// LOC100046031	17.24	17.27
FXYD domain-containing ion transport regulator 7	Fxyd7	17.14	52.15
transcription factor-like 5 (basic helix-loop-helix)	Tcfl5	16.43	26.69
testis expressed gene 15	Tex15	16.33	19.61
similar to Tescalcin /// tescalcin	LOC100047138 /// Tesc	13.81	32.22
desert hedgehog	Dhh	12.84	17.21
insulin-like 6	Insl6	12.82	18.6
receptor accessory protein 6	Reep6	12.23	4.92
heat shock protein 1B	Hspa1b	12.11	3.3
nidogen 2	Nid2	11.44	5.77
KDEL (Lys-Asp-Glu-Leu) endoplasmic reticulum protein retention receptor 3	Kdelr3	11.15	6.87
thioredoxin interacting protein	Txnip	8.68	3.75
zinc finger, CCHC domain containing 12	Zcchc12	8.5	9.35
Kruppel-like factor 4 (gut)	Klf4	7.94	4.74
G protein regulated inducer of neurite outgrowth 2	Gprin2	7.51	6.34
major facilitator superfamily domain containing 7C	Mfsd7c	7.43	6.84
ELL associated factor 2	Eaf2	6.98	9.88
phorbol-12-myristate-13-acetate-induced protein 1	Pmaip1	6.82	5.9
rhophilin, Rho GTPase binding protein 2	Rhpn2	6.75	3.71
somatostatin receptor 2	Sstr2	6.25	5.43
biliverdin reductase B (flavin reductase (NADPH))	Blvrb	6.17	6.73
BH3 interacting domain death agonist	Bid	5.92	3.31
Rho GTPase activating protein 29	Arhgap29	5.84	4.35
grancalcin	Gca	5.82	4.31
diacylglycerol kinase kappa	Dgkk	5.66	16.7
opioid growth factor receptor-like 1	Ogfrl1	5.59	3.58
six transmembrane epithelial antigen of the prostate 1	Steap1	5.51	7.7
histocompatibility 2, D region locus 1 /// histocompatibility 2, K1, K region /// similar to H-2K(d) antigen	H2-D1 /// H2-K1 /// LOC100044874	5.45	6.67
fatty acid desaturase 3	Fads3	5.36	6.04
WD repeat domain 17	Wdr17	5.36	3.26
mitogen-activated protein kinase-activated protein kinase 3	Mapkapk3	5.32	4.2
sodium channel, voltage-gated, type III, beta	Scn3b	5	5.57
major facilitator superfamily domain containing 9	Mfsd9	4.95	4.73
paraneoplastic antigen MA2	Pnma2	4.88	5.8
chromobox homolog 7	Cbx7	4.73	4.74

continue

Table S10 (Part 2)

Regulated Genes TSA 6h and TSA 24h	Symbol	Fold Change TSA 6h	Fold Change TSA 24h
phosphatidylinositol glycan anchor biosynthesis, class F	Pigf	4.55	2.26
histocompatibility 2, K1, K region	H2-K1	4.53	7.03
B-cell translocation gene 3 /// B-cell translocation gene 3 pseudogene	Btg3 /// Gm7334	4.45	3.81
shroom family member 3	Shroom3	4.32	5.18
spermatogenesis associated 6	Spata6	4.31	3.92
uveal autoantigen with coiled-coil domains and ankyrin repeats	Uaca	4.29	4.76
RAB3D, member RAS oncogene family	Rab3d	4.23	6.19
lectin, galactose binding, soluble 1	Lgals1	4.21	7.12
cytochrome c oxidase subunit VIb polypeptide 2	Cox6b2	4.03	9.24
dual-specificity tyrosine-(Y)-phosphorylation regulated kinase 3	Dyrk3	3.93	3.75
cytochrome P450, family 39, subfamily a, polypeptide 1	Cyp39a1	3.75	6.68
hepatic leukemia factor	Hlf	3.74	5.58
solute carrier family 2 (facilitated glucose transporter), member 3	Slc2a3	3.71	3.31
integral membrane protein 2A	Itm2a	3.7	7.38
cytochrome b5 reductase 1	Cyb5r1	3.68	2.61
hippocalcin-like 1	Hpcal1	3.61	3.83
LPS-induced TN factor	Litaf	3.6	3.32
A kinase (PRKA) anchor protein (gravin) 12	Akap12	3.58	5.08
cysteine and glycine-rich protein 2	Csrp2	3.56	6.06
NHL repeat containing 1	Nhlrc1	3.56	2.46
SH3 domain binding glutamic acid-rich protein like 2	Sh3bgrl2	3.54	3.12
serine (or cysteine) peptidase inhibitor, clade I, member 1	Serpini1	3.53	2.52
CD44 antigen	Cd44	3.49	3.56
melanoma associated antigen (mutated) 1-like 1	Mum1l1	3.49	3.09
immunoglobulin superfamily, member 9	Igsf9	3.48	3.71
histocompatibility 2, D region locus 1	H2-D1	3.44	6.16
tumor necrosis factor, alpha-induced protein 8	Tnfaip8	3.43	5.17
cellular repressor of E1A-stimulated genes 1	Creg1	3.42	2.34
ATP-binding cassette, sub-family A (ABC1), member 5	Abca5	3.38	2.83
ARP3 actin-related protein 3 homolog B (yeast)	Actr3b	3.32	3.23
thymus cell antigen 1, theta	Thy1	3.3	9.99
lactamase, beta 2	Lactb2	3.27	2.89
aquaporin 11	Aqp11	3.24	4.61
EF hand domain containing 1	Efhd1	3.22	6.77
small nucleolar RNA, C/D box 123	Snord123	3.22	10.37
regulator of G-protein signalling 10	Rgs10	3.2	6.39
fucosyltransferase 10	Fut10	3.16	2.38
solute carrier family 29 (nucleoside transporters), member 1	Slc29a1	3.15	3.79
ribosomal protein S6 kinase polypeptide 1	Rps6ka1	3.14	2.37

continue

Table S10 (Part 3)

Regulated Genes TSA 6h and TSA 24h	Symbol	Fold Change TSA 6h	Fold Change TSA 24h
phosphatidylinositol transfer protein, membrane-associated 1	Pitpnm1	3.14	3.5
PDZ and LIM domain 7	Pdlim7	3.11	2.48
sarcalumenin	Srl	3.11	2.48
anterior pharynx defective 1b homolog (C, elegans) /// anterior pharynx defective 1c homolog (C, elegans)	Aph1b /// Aph1c	3.03	3.12
transmembrane protein 218	Tmem218	2.99	3.07
histocompatibility 2, D region	H2-L	2.95	5.24
scavenger receptor class B, member 1	Scarb1	2.93	2.39
Tmsb15b1-Tmsb15b2 readthrough transcript /// thymosin beta 15b2	Tmsb15b1-Tmsb15b2 /// Tmsb15b2	2.91	3.41
X-linked myotubular myopathy gene 1	Mtm1	2.89	2.65
PRKC, apoptosis, WT1, regulator	Pawr	2.89	2.71
cDNA sequence X99384	X99384	2.88	4.05
pituitary tumor-transforming gene 1	Pttg1	2.87	2.37
microsomal glutathione S-transferase 3	Mgst3	2.87	3.67
zinc finger protein 217	Zfp217	2.83	2.65
sorbitol dehydrogenase	Sord	2.78	4.11
G protein-coupled receptor associated sorting protein 2	Gprasp2	2.78	2.41
transmembrane protein 41a	Tmem41a	2.68	2.94
EGL nine homolog 3 (C, elegans)	Egln3	2.64	3.5
cysteine conjugate-beta lyase 2	Ccbl2	2.64	3.26
vesicle amine transport protein 1 homolog-like (T, californica)	Vat1l	2.64	2.67
synaptonemal complex central element protein 2	Syce2	2.55	3.62
calcium binding atopy-related autoantigen 1	Cbara1	2.49	2.58
prolylcarboxypeptidase (angiotensinase C)	Prcp	2.45	4.56
unc-13 homolog B (C, elegans)	Unc13b	2.37	3.12
frizzled homolog 7 (Drosophila)	Fzd7	2.36	3
SEH1-like (S, cerevisiae	Seh1l	-2.18	-2.37
scrapie responsive gene 1	Scrg1	-2.21	-2.94
poly(rC) binding protein 4	Pcbp4	-2.34	-2.51
cytohesin 2	Cyth2	-2.34	-2.68
BR serine/threonine kinase 1	Brsk1	-2.34	-3.09
ubiquitin-fold modifier conjugating enzyme 1	Ufc1	-2.35	-3.35
Kv channel interacting protein 3, calsenilin	Kcnip3	-2.4	-2.81
proviral integration site 3	Pim3	-2.41	-5.92
glycerol-3-phosphate dehydrogenase 1 (soluble)	Gpd1	-2.5	-7.87
KN motif and ankyrin repeat domains 1	Kank1	-2.51	-3.29
protein tyrosine phosphatase, receptor type, E	Ptpre	-2.53	-7.19
cytoplasmic FMR1 interacting protein 2	Cyfip2	-2.53	-3.79
kelch-like 2, Mayven (Drosophila)	Klhl2	-2.56	-4.08
dedicator of cytokinesis 9	Dock9	-2.63	-3.28
ring finger protein 122	Rnf122	-2.63	-2.92

continue

Table S10 (Part 4)

Regulated Genes TSA 6h and TSA 24h	Symbol	Fold Change TSA 6h	Fold Change TSA 24h
B-cell CLL/lymphoma 7A	Bcl7a	-2.64	-2.85
chromodomain helicase DNA binding protein 3	Chd3	-2.8	-4.41
SRY-box containing gene 10	Sox10	-2.81	-13.51
LAG1 homolog, ceramide synthase 2	Lass2	-2.82	-2.77
lysophosphatidylglycerol acyltransferase 1	Lpgat1	-2.87	-6.43
poly (ADP-ribose) polymerase family, member 8	Parp8	-2.88	-2.76
transmembrane and coiled-coil domains 2	Tmcc2	-2.9	-5.09
BR serine/threonine kinase 2	Brsk2	-2.9	-2.47
Rho guanine nucleotide exchange factor (GEF7)	Arhgef7	-2.91	-5.52
myeloid/lymphoid or mixed-lineage leukemia (trithorax homolog, Drosophila); translocated to, 11	Mllt11	-2.92	-5.38
NAC alpha domain containing	Nacad	-2.92	-2.81
striatin, calmodulin binding protein	Strn	-2.93	-4.04
breast carcinoma amplified sequence 1	Bcas1	-2.93	-13.89
RUN and SH3 domain containing 2	Rusc2	-2.94	-4.28
ectonucleotide pyrophosphatase/phosphodiesterase 6	Enpp6	-2.98	-33.26
Down syndrome cell adhesion molecule	Dscam	-2.98	-4.57
S100 calcium binding protein A16	S100a16	-2.99	-2.69
family with sequence similarity 19, member A2	Fam19a2	-3.1	-5.38
discs, large (Drosophila) homolog-associated protein 1	Dlgap1	-3.19	-2.42
Down syndrome cell adhesion molecule-like 1	Dscaml1	-3.19	-5.91
nibrin	Nbn	-3.25	-2.75
low density lipoprotein receptor	Ldlr	-3.31	-13.49
TAF9B RNA polymerase II, TATA box binding protein (TBP)-associated factor	Taf9b	-3.31	-2.63
delta-like 3 (Drosophila)	Dll3	-3.32	-4.49
ankyrin 3, epithelial	Ank3	-3.32	-3.88
ELOVL family member 7, elongation of long chain fatty acids (yeast)	Elovl7	-3.33	-14.34
kit oncogene	Kit	-3.39	-8.77
doublecortin	Dcx	-3.45	-10.12
phosphodiesterase 4B, cAMP specific	Pde4b	-3.48	-3.39
S100 protein, beta polypeptide, neural	S100b	-3.54	-14.71
WAS/WASL interacting protein family, member 1	Wipf1	-3.64	-4.68
collagen, type IX, alpha 1	Col9a1	-3.69	-6.2
shroom family member 2	Shroom2	-3.69	-4.46
phosphatidic acid phosphatase type 2 domain containing 1B	Ppapdc1b	-3.74	-3.85
solute carrier family 24 (sodium/potassium/calcium exchanger), member 3	Slc24a3	-3.79	-4.96
mex3 homolog B (C, elegans)	Mex3b	-3.79	-2.77
uronyl-2-sulfotransferase	Ust	-3.79	-4.11
retinaldehyde binding protein 1	Rlbp1	-3.81	-3.14
hydroxy-delta-5-steroid dehydrogenase, 3 beta- and steroid delta-isomerase 7	Hsd3b7	-3.95	-6.8

continue

Table S10 (Part 5)

Regulated Genes TSA 6h and TSA 24h	Symbol	Fold Change TSA 6h	Fold Change TSA 24h
plexin B3	Plxnb3	-4.09	-19.44
predicted gene 98	Gm98	-4.13	-20.43
beaded filament structural protein 2, phakinin	Bfsp2	-4.14	-6.58
minichromosome maintenance deficient 3 (S, cerevisiae) associated protein	Mcm3ap	-4.24	-2.63
predicted gene 13111	Gm13111	-4.3	-3.66
ligand dependent nuclear receptor corepressor-like	Lcorl	-4.32	-12.53
versican	Vcan	-4.37	-4.57
MAP6 domain containing 1	Map6d1	-4.51	-17.39
regulator of chromosome condensation (RCC1) and BTB (POZ) domain containing protein 2	Rcbtb2	-4.52	-3.94
platelet derived growth factor, alpha	Pdgfa	-4.54	-3.59
adaptor-related protein complex AP-4, sigma 1	Ap4s1	-4.6	-3.48
LIM and senescent cell antigen like domains 2	Lims2	-4.61	-27.27
leucine rich repeat containing 4	Lrrc4	-4.72	-2.47
tubulin, beta 4	Tubb4	-4.74	-12.31
actin filament associated protein 1-like 2	Afap1l2	-4.84	-4.16
H2A histone family, member Y2 /// H2A histone family, member Y3	H2afy2 /// H2afy3	-4.88	-3.89
family with sequence similarity 176, member A	Fam176a	-4.89	-3.96
galactose-3-O-sulfotransferase 1	Gal3st1	-4.96	-18.2
chimerin (chimaerin) 2	Chn2	-5.09	-7.68
tumor suppressor candidate 1	Tusc1	-5.46	-2.79
zinc finger E-box binding homeobox 2	Zeb2	-5.52	-3.15
serine/arginine-rich protein specific kinase 3	Srpk3	-6.01	-26.6
G protein-coupled receptor 149	Gpr149	-6.26	-18.02
RNA binding motif protein, X chromosome	Rbmx	-6.29	-3.7
DMRT-like family B with proline-rich C-terminal, 1	Dmrtb1	-6.29	-5.7
suppression of tumorigenicity 18	St18	-6.44	-30.35
potassium channel tetramerisation domain containing 4	Kctd4	-6.66	-14.35
G0/G1 switch gene 2	G0s2	-6.74	-4.02
proline rich region 18	Prr18	-6.74	-4.09
four and a half LIM domains 2	Fhl2	-6.79	-9.48
G protein-coupled receptor 17	Gpr17	-6.8	-80.21
RAB33A, member of RAS oncogene family	Rab33a	-7.1	-13.66
leucine rich repeat transmembrane neuronal 1	Lrrtm1	-7.54	-5.2
suppressor of variegation 4-20 homolog 1 (Drosophila)	Suv420h1	-8.05	-3.29
apoptosis, caspase activation inhibitor	Aven	-8.33	-4.14
apoptosis-associated tyrosine kinase	Aatk	-9.6	-17.1
NK2 transcription factor related, locus 2 (Drosophila)	Nkx2-2	-11.28	-17.97
oligodendrocyte myelin glycoprotein	Omg	-11.48	-9.86
protein kinase C, theta	Prkcq	-11.82	-12.45
H2A histone family, member Y2	H2afy2	-14.59	-6.17

Table S11:
GO Terms obtained from the of hierarchical Cluster Analysis (Fig. 3G, Genes 1-200) for the Sub-cluster:
TSA up-regulated 6h

GO Term	Count	p-Value
GO:0031090~organelle membrane	15	1.08E-03

Table S12:
GO Terms obtained from the of hierarchical Cluster Analysis (Fig. 3G, Genes 201-428) for the Sub-cluster:
TSA up-regulated 6h & 24h

GO Term	Count	p-Value
GO:0044464~cell part	145	2.01E-04
GO:0005623~cell	145	2.01E-04

Table S13:
GO Terms obtained from the of hierarchical Cluster Analysis (Fig. 3G, Genes 428-789) for the Sub-cluster:
TSA up-regulated 24h

GO Term	Count	p-Value
GO:0042611~MHC protein complex	10	4.52E-08
GO:0048002~antigen processing and presentation of peptide antigen	8	1.81E-06
GO:0019882~antigen processing and presentation	10	4.04E-06
GO:0042612~MHC class I protein complex	7	1.69E-05
GO:0008289~lipid binding	19	1.23E-04
GO:0048503~GPI anchor binding	10	1.26E-04
GO:0002474~antigen processing and presentation of peptide antigen via MHC class I	5	2.65E-04
GO:0005543~phospholipid binding	13	3.74E-04
GO:0050896~response to stimulus	48	4.52E-04
GO:0004364~glutathione transferase activity	5	4.81E-04
GO:0044444~cytoplasmic part	80	5.12E-04
GO:0005507~copper ion binding	7	7.13E-04
GO:0005576~extracellular region	61	9.70E-04
GO:0005886~plasma membrane	48	1.06E-03

Table S14:
GO Terms obtained from the of hierarchical Cluster Analysis (Fig. 3G, genes 1-789) for the Sub-cluster:
TSA up-regulated

GO Term	Count	p-Value
GO:0042611~MHC protein complex	10	1.86E-05
GO:0004364~glutathione transferase activity	7	9.99E-05
GO:0048002~antigen processing and presentation of peptide antigen	8	2.48E-04
GO:0005737~cytoplasm	237	3.01E-04
GO:0044444~cytoplasmic part	150	3.67E-04
GO:0048503~GPI anchor binding	13	7.11E-04
GO:0048037~cofactor binding	17	7.65E-04
GO:0042612~MHC class I protein complex	7	8.86E-04

Table S15:
GO Terms obtained from the of hierarchical Cluster Analysis (Fig. 3G, genes 1799-2074) for the Sub-cluster:
TSA down-regulated

GO Term	Count	p-Value
GO:0006323~DNA packaging	20	7.18E-10
GO:0006325~establishment and/or maintenance of chromatin architecture	19	3.15E-09
GO:0016569~covalent chromatin modification	10	1.10E-08
GO:0016568~chromatin modification	16	1.24E-08
GO:0043283~biopolymer metabolic process	94	1.31E-08
GO:0044427~chromosomal part	19	3.62E-08
GO:0005694~chromosome	20	5.70E-08
GO:0051276~chromosome organization and biogenesis	19	2.06E-07
GO:0005634~nucleus	83	2.47E-06
GO:0016573~histone acetylation	5	8.39E-06
GO:0000785~chromatin	12	1.04E-05
GO:0003676~nucleic acid binding	68	1.18E-05
GO:0006259~DNA metabolic process	23	1.89E-05
GO:0016570~histone modification	7	2.33E-05
GO:0065004~protein-DNA complex assembly	9	2.49E-05
GO:0008134~transcription factor binding	14	2.94E-05
GO:0031497~chromatin assembly	8	3.60E-05
GO:0050794~regulation of cellular process	73	3.62E-05
GO:0016070~RNA metabolic process	56	3.80E-05
GO:0006473~protein amino acid acetylation	5	4.40E-05
GO:0031981~nuclear lumen	25	5.02E-05
GO:0006333~chromatin assembly or disassembly	9	6.05E-05
GO:0005654~nucleoplasm	22	6.65E-05
GO:0044428~nuclear part	31	7.49E-05
GO:0043170~macromolecule metabolic process	102	8.40E-05
GO:0044451~nucleoplasm part	21	8.42E-05
GO:0006139~nucleobase, nucleoside, nucleotide and nucleic acid metabolic process	66	8.44E-05
GO:0019219~regulation of nucleobase, nucleoside, nucleotide and nucleic acid metabolic process	50	8.57E-05
GO:0006334~nucleosome assembly	7	9.12E-05
GO:0006355~regulation of transcription, DNA-dependent	47	1.42E-04
GO:0050789~regulation of biological process	76	1.64E-04
GO:0006351~transcription, DNA-dependent	47	1.89E-04
GO:0032774~RNA biosynthetic process	47	1.98E-04
GO:0043234~protein complex	41	2.09E-04
GO:0045449~regulation of transcription	48	2.24E-04
GO:0006350~transcription	49	2.53E-04
GO:0010468~regulation of gene expression	50	2.77E-04
GO:0031974~membrane-enclosed lumen	25	3.20E-04
GO:0043233~organelle lumen	25	3.20E-04
GO:0043543~protein amino acid acylation	5	3.27E-04
GO:0043687~post-translational protein modification	34	3.57E-04

continue

Table S15 (Part 2)

GO Term	Count	p-Value
GO:0031323~regulation of cellular metabolic process	51	3.83E-04
GO:0005515~protein binding	100	4.73E-04
GO:0006464~protein modification process	37	5.16E-04
GO:0043412~biopolymer modification	38	5.28E-04
GO:0065003~macromolecular complex assembly	14	6.03E-04
GO:0000122~negative regulation of transcription from RNA polymerase II promoter	9	7.80E-04
GO:0022607~cellular component assembly	15	8.59E-04
GO:0032991~macromolecular complex	45	9.58E-04
GO:0019222~regulation of metabolic process	51	1.00E-03

Table S16:
GO Terms obtained from the of hierarchical Cluster Analysis (Fig. 3G, genes 790-878) for the Sub-cluster:
TSA & BMP2 up-regulated

GO Term	Count	p-Value
GO:0007167~enzyme linked receptor protein signaling pathway	10	2.63E-05
GO:0005539~glycosaminoglycan binding	6	7.79E-05
GO:0030247~polysaccharide binding	6	1.17E-04
GO:0007155~cell adhesion	13	1.54E-04
GO:0022610~biological adhesion	13	1.54E-04
GO:0001871~pattern binding	6	1.62E-04
GO:0008201~heparin binding	5	3.81E-04
GO:0007169~transmembrane receptor protein tyrosine kinase signaling pathway	7	4.93E-04
GO:0005578~proteinaceous extracellular matrix	8	6.20E-04
GO:0031012~extracellular matrix	8	7.00E-04
GO:0007160~cell-matrix adhesion	5	7.76E-04
GO:0031589~cell-substrate adhesion	5	9.33E-04

Table S17:
GO Terms obtained from the of hierarchical Cluster Analysis (Fig. 3G, genes 1283-1798) for the Sub-cluster:
TSA & BMP2 down-regulated

GO Term	Count	p-Value
GO:0007399~nervous system development	46	1.42E-12
GO:0032502~developmental process	106	8.22E-10
GO:0048468~cell development	56	2.81E-08
GO:0048856~anatomical structure development	78	3.53E-08
GO:0022008~neurogenesis	27	6.14E-08
GO:0008092~cytoskeletal protein binding	28	6.43E-08
GO:0048731~system development	69	8.25E-08
GO:0030154~cell differentiation	71	1.40E-07
GO:0048869~cellular developmental process	71	1.40E-07
GO:0007275~multicellular organismal development	80	3.94E-07
GO:0043209~myelin sheath	5	9.74E-07
GO:0032501~multicellular organismal process	94	1.64E-06

continue

Table S17 (Part 2)

GO Term	Count	p-Value
GO:0001508~regulation of action potential	9	2.79E-06
GO:0007272~ensheathment of neurons	8	4.02E-06
GO:0008366~axon ensheathment	8	4.02E-06
GO:0005515~protein binding	159	5.17E-06
GO:0048699~generation of neurons	22	9.79E-06
GO:0030182~neuron differentiation	20	1.89E-05
GO:0003779~actin binding	19	2.18E-05
GO:0005856~cytoskeleton	39	2.50E-05
GO:0042552~myelination	7	3.52E-05
GO:0050877~neurological system process	27	5.73E-05
GO:0007154~cell communication	87	6.01E-05
GO:0019226~transmission of nerve impulse	17	9.22E-05
GO:0007267~cell-cell signaling	21	9.26E-05
GO:0000902~cell morphogenesis	26	9.54E-05
GO:0032989~cellular structure morphogenesis	26	9.54E-05
GO:0030323~respiratory tube development	9	1.03E-04
GO:0007409~axonogenesis	13	1.92E-04
GO:0007010~cytoskeleton organization and biogenesis	24	2.68E-04
GO:0016020~membrane	156	2.88E-04
GO:0009987~cellular process	236	3.68E-04
GO:0048812~neurite morphogenesis	13	3.82E-04
GO:0048667~neuron morphogenesis during differentiation	13	3.82E-04
GO:0030030~cell projection organization and biogenesis	17	4.09E-04
GO:0048858~cell projection morphogenesis	17	4.09E-04
GO:0032990~cell part morphogenesis	17	4.09E-04
GO:0031175~neurite development	14	4.88E-04
GO:0030324~lung development	8	5.88E-04
GO:0017048~Rho GTPase binding	5	9.20E-04
GO:0005623~cell	276	9.31E-04
GO:0044464~cell part	276	9.31E-04
GO:0045843~negative regulation of striated muscle development	4	9.42E-04
GO:0005886~plasma membrane	56	9.56E-04
GO:0006915~apoptosis	28	1.05E-03

Table S18:
GO Terms obtained from the of hierarchical Cluster Analysis (Fig. 3G, genes 959-1090) for the Sub-cluster:
BMP2 up-regulated 6h & 24h

GO Term	Count	p-Value
GO:0007167~enzyme linked receptor protein signaling pathway	15	2.47E-09
GO:0007178~transmembrane receptor protein serine/threonine kinase signaling pathway	8	8.23E-07
GO:0007154~cell communication	36	2.68E-06
GO:0007166~cell surface receptor linked signal transduction	23	5.40E-06
GO:0007165~signal transduction	33	9.44E-06
GO:0007275~multicellular organismal development	30	1.28E-05
GO:0048856~anatomical structure development	28	2.05E-05
GO:0048731~system development	25	3.76E-05
GO:0030509~BMP signaling pathway	5	4.06E-05
GO:0032502~developmental process	34	5.98E-05
GO:0030154~cell differentiation	25	9.26E-05
GO:0048869~cellular developmental process	25	9.26E-05
GO:0032501~multicellular organismal process	31	6.00E-04

Table S19:
GO Terms obtained from the of hierarchical Cluster Analysis (Fig. 3G, genes 1091-1282) for the Sub-cluster:
BMP2 up-regulated 24h

GO Term	Count	p-Value
GO:0007275~multicellular organismal development	39	2.23E-06
GO:0032502~developmental process	44	2.26E-05
GO:0032501~multicellular organismal process	42	7.00E-05
GO:0009887~organ morphogenesis	15	1.17E-04
GO:0048514~blood vessel morphogenesis	9	2.56E-04
GO:0048468~cell development	23	3.02E-04
GO:0048513~organ development	25	3.13E-04
GO:0009653~anatomical structure morphogenesis	22	4.63E-04
GO:0030154~cell differentiation	29	5.42E-04
GO:0048869~cellular developmental process	29	5.42E-04
GO:0007154~cell communication	39	5.46E-04
GO:0001568~blood vessel development	9	6.92E-04
GO:0001944~vasculature development	9	7.51E-04
GO:0007166~cell surface receptor linked signal transduction	23	8.80E-04
GO:0007507~heart development	8	8.83E-04
GO:0016477~cell migration	10	8.94E-04

Table S20:
GO Terms obtained from the of hierarchical Cluster Analysis (Fig. 3G, genes 595-1282) for the Sub-cluster:
BMP2 up-regulated

GO Term	Count	p-Value
GO:0007275~multicellular organismal development	68	3.96E-11
GO:0007167~enzyme linked receptor protein signaling pathway	22	1.16E-09
GO:0032502~developmental process	76	4.61E-09
GO:0007154~cell communication	73	1.16E-08
GO:0030154~cell differentiation	54	4.45E-08
GO:0048869~cellular developmental process	54	4.45E-08
GO:0007166~cell surface receptor linked signal transduction	44	5.83E-08
GO:0032501~multicellular organismal process	72	6.00E-08
GO:0048731~system development	51	1.40E-07
GO:0048856~anatomical structure development	55	5.42E-07
GO:0007165~signal transduction	64	8.18E-07
GO:0007507~heart development	14	2.06E-06
GO:0009887~organ morphogenesis	23	4.91E-06
GO:0048513~organ development	41	5.38E-06
GO:0009653~anatomical structure morphogenesis	36	9.75E-06
GO:0048514~blood vessel morphogenesis	13	2.50E-05
GO:0007178~transmembrane receptor protein serine/threonine kinase signaling pathway	9	2.67E-05
GO:0007399~nervous system development	24	4.06E-05
GO:0048468~cell development	35	5.64E-05
GO:0001568~blood vessel development	13	1.06E-04
GO:0001944~vasculature development	13	1.20E-04
GO:0016477~cell migration	14	3.30E-04
GO:0007169~transmembrane receptor protein tyrosine kinase signaling pathway	11	3.38E-04
GO:0009968~negative regulation of signal transduction	8	4.97E-04
GO:0048519~negative regulation of biological process	28	9.44E-04
GO:0030509~BMP signaling pathway	5	1.02E-03
GO:0051216~cartilage development	6	1.07E-03

Table S21:
GO Terms obtained from the of hierarchical Cluster Analysis (Fig. 3G, genes 878-959) for the Sub-cluster:
BMP2 down-regulated

GO Term	Count	p-Value
GO:0007165~signal transduction	23	4.22E-04
GO:0007154~cell communication	24	5.91E-04

Table S22:
Significant GO Terms from functional Annotation Cluster (Fig. 4C) of all Genes regulated within following Treatment: **BMP2 6h**

GO Term	Count	p-Value
GO:0007154~cell communication	63	4.80E-12
GO:0007167~enzyme linked receptor protein signaling pathway	18	5.46E-09
GO:0007165~signal transduction	52	5.85E-08
GO:0007166~cell surface receptor linked signal transduction	33	7.60E-07
GO:0007178~transmembrane receptor protein serine/threonine kinase signaling pathway	9	2.00E-06
GO:0007389~pattern specification process	12	1.78E-05
GO:0007399~nervous system development	20	2.01E-05
GO:0007275~multicellular organismal development	41	3.74E-05
GO:0048731~system development	34	7.56E-05
GO:0003002~regionalization	9	8.66E-05
GO:0048514~blood vessel morphogenesis	10	1.62E-04
GO:0030154~cell differentiation	34	2.21E-04
GO:0048869~cellular developmental process	34	2.21E-04
GO:0065007~biological regulation	63	2.45E-04
GO:0030509~BMP signaling pathway	5	2.72E-04
GO:0048856~anatomical structure development	36	3.21E-04
GO:0048519~negative regulation of biological process	23	3.66E-04
GO:0050789~regulation of biological process	58	4.85E-04
GO:0001568~blood vessel development	10	4.89E-04
GO:0001944~vasculature development	10	5.36E-04
GO:0032502~developmental process	46	5.43E-04
GO:0032501~multicellular organismal process	45	6.12E-04
GO:0050793~regulation of developmental process	11	7.29E-04
GO:0009888~tissue development	11	7.86E-04
GO:0048513~organ development	27	8.46E-04
GO:0051093~negative regulation of developmental process	7	9.31E-04
GO:0001525~angiogenesis	8	9.35E-04
GO:0048523~negative regulation of cellular process	21	9.82E-04

Table S23:
Significant GO Terms from functional Annotation Cluster (Fig. 4D) of all Genes regulated within following Treatment: **BMP2 24h**

GO Term	Count	p-Value
GO:0007275~multicellular organismal development	113	3.13E-14
GO:0032502~developmental process	135	6.54E-14
GO:0007154~cell communication	127	2.93E-12
GO:0048856~anatomical structure development	101	5.72E-12
GO:0009653~anatomical structure morphogenesis	70	2.09E-11
GO:0048731~system development	88	9.15E-11
GO:0007165~signal transduction	115	1.08E-10
GO:0007167~enzyme linked receptor protein signaling pathway	31	1.78E-10
GO:0007166~cell surface receptor linked signal transduction	72	5.86E-10
GO:0032501~multicellular organismal process	120	2.45E-09
GO:0007399~nervous system development	45	4.12E-09
GO:0009887~organ morphogenesis	38	3.37E-08
GO:0030154~cell differentiation	84	3.92E-08
GO:0048869~cellular developmental process	84	3.92E-08
GO:0048514~blood vessel morphogenesis	22	5.01E-08
GO:0016020~membrane	199	1.32E-07
GO:0001568~blood vessel development	23	1.51E-07
GO:0001944~vasculature development	23	1.88E-07
GO:0007507~heart development	19	1.46E-06
GO:0048646~anatomical structure formation	20	2.00E-06
GO:0044425~membrane part	162	2.73E-06
GO:0001525~angiogenesis	17	3.03E-06
GO:0000902~cell morphogenesis	33	3.75E-06
GO:0032989~cellular structure morphogenesis	33	3.75E-06
GO:0048513~organ development	64	4.11E-06
GO:0048468~cell development	58	4.55E-06
GO:0005515~protein binding	182	8.76E-06
GO:0007411~axon guidance	12	1.12E-05
GO:0016477~cell migration	23	1.18E-05
GO:0007178~transmembrane receptor protein serine/threonine kinase signaling pathway	12	1.25E-05
GO:0022610~biological adhesion	37	1.62E-05
GO:0007155~cell adhesion	37	1.62E-05
GO:0030030~cell projection organization and biogenesis	22	1.78E-05
GO:0048858~cell projection morphogenesis	22	1.78E-05
GO:0032990~cell part morphogenesis	22	1.78E-05
GO:0060089~molecular transducer activity	72	2.55E-05
GO:0004871~signal transducer activity	72	2.55E-05
GO:0022008~neurogenesis	25	2.66E-05
GO:0031175~neurite development	18	2.80E-05
GO:0048812~neurite morphogenesis	16	5.39E-05
GO:0048667~neuron morphogenesis during differentiation	16	5.39E-05
GO:0004872~receptor activity	62	8.49E-05

continue

Table S23 (Part 2)

GO Term	Count	p-Value
GO:0003002~regionalization	14	8.74E-05
GO:0007409~axonogenesis	15	8.98E-05
GO:0030182~neuron differentiation	21	9.34E-05
GO:0007389~pattern specification process	18	1.07E-04
GO:0007169~transmembrane receptor protein tyrosine kinase signaling pathway	16	1.18E-04
GO:0048666~neuron development	18	1.31E-04
GO:0030509~BMP signaling pathway	7	1.53E-04
GO:0006928~cell motility	23	1.61E-04
GO:0051674~localization of cell	23	1.61E-04
GO:0016021~integral to membrane	139	1.62E-04
GO:0048699~generation of neurons	22	1.78E-04
GO:0031224~intrinsic to membrane	139	2.03E-04
GO:0000904~cellular morphogenesis during differentiation	16	2.06E-04
GO:0048754~branching morphogenesis of a tube	9	2.16E-04
GO:0048518~positive regulation of biological process	46	2.51E-04
GO:0001763~morphogenesis of a branching structure	9	3.63E-04
GO:0051094~positive regulation of developmental process	10	3.98E-04
GO:0030323~respiratory tube development	9	4.00E-04
GO:0048522~positive regulation of cellular process	41	4.67E-04
GO:0021532~neural tube patterning	4	5.16E-04
GO:0051216~cartilage development	8	5.44E-04
GO:0019898~extrinsic to membrane	9	7.98E-04
GO:0002062~chondrocyte differentiation	5	9.18E-04

Table S24:
Significant GO Terms from functional Annotation Cluster (Fig. 4A) of all Genes regulated within following Treatment: **TSA 6h**

GO Term	Count	p-Value
GO:0016569~covalent chromatin modification	12	2.07E-06
GO:0032502~developmental process	158	6.71E-06
GO:0016573~histone acetylation	6	3.29E-05
GO:0030154~cell differentiation	105	3.30E-05
GO:0048869~cellular developmental process	105	3.30E-05
GO:0006325~establishment and/or maintenance of chromatin architecture	25	3.37E-05
GO:0005515~protein binding	270	3.61E-05
GO:0006323~DNA packaging	25	5.30E-05
GO:0006464~protein modification process	92	7.20E-05
GO:0016568~chromatin modification	20	7.88E-05
GO:0005623~cell	510	9.77E-05
GO:0044464~cell part	510	9.77E-05
GO:0043412~biopolymer modification	94	1.12E-04
GO:0051276~chromosome organization and biogenesis	28	1.58E-04
GO:0006915~apoptosis	47	2.21E-04
GO:0043687~post-translational protein modification	80	2.40E-04
GO:0006473~protein amino acid acetylation	6	2.65E-04
GO:0048468~cell development	73	2.79E-04
GO:0016570~histone modification	9	2.97E-04
GO:0043283~biopolymer metabolic process	211	3.12E-04
GO:0012501~programmed cell death	47	3.25E-04
GO:0006996~organelle organization and biogenesis	67	3.29E-04
GO:0043543~protein amino acid acylation	7	3.95E-04
GO:0008219~cell death	47	7.21E-04
GO:0016265~death	47	7.21E-04

Table S25:
Significant GO Terms from functional Annotation Cluster (Fig. 4B) of all Genes regulated within following Treatment: **TSA 24h**

GO Term	Count	p-Value
GO:0032502~developmental process	180	6.66E-08
GO:0005886~plasma membrane	118	1.05E-06
GO:0007155~cell adhesion	56	2.16E-06
GO:0022610~biological adhesion	56	2.16E-06
GO:0030154~cell differentiation	117	4.52E-06
GO:0048869~cellular developmental process	117	4.52E-06
GO:0048468~cell development	85	8.08E-06
GO:0016020~membrane	318	8.10E-06
GO:0048856~anatomical structure development	122	5.11E-05
GO:0000902~cell morphogenesis	43	6.33E-05
GO:0032989~cellular structure morphogenesis	43	6.33E-05
GO:0042611~MHC protein complex	10	9.61E-05
GO:0007399~nervous system development	50	1.26E-04
GO:0048731~system development	105	1.51E-04
GO:0007275~multicellular organismal development	127	3.39E-04
GO:0044459~plasma membrane part	84	4.80E-04
GO:0005539~glycosaminoglycan binding	13	4.91E-04
GO:0048002~antigen processing and presentation of peptide antigen	8	8.15E-04
GO:0043209~myelin sheath	4	8.29E-04

Table S26:
Significant GO Terms from functional Annotation Cluster (Fig. 4E) of all Genes regulated within following Treatment: **BMP2 24h and TSA 24h**

GO Term	Count	p-Value
GO:0048856~anatomical structure development	35	1.04E-06
GO:0000902~cell morphogenesis	17	1.13E-06
GO:0032989~cellular structure morphogenesis	17	1.13E-06
GO:0007167~enzyme linked receptor protein signaling pathway	13	2.13E-06
GO:0032502~developmental process	42	6.34E-06
GO:0009653~anatomical structure morphogenesis	24	9.55E-06
GO:0007154~cell communication	40	1.57E-05
GO:0007399~nervous system development	17	2.31E-05
GO:0016020~membrane	65	2.51E-05
GO:0042325~regulation of phosphorylation	6	1.44E-04
GO:0007275~multicellular organismal development	32	1.63E-04
GO:0019220~regulation of phosphate metabolic process	6	1.76E-04
GO:0051174~regulation of phosphorus metabolic process	6	1.76E-04
GO:0007165~signal transduction	35	1.97E-04
GO:0048731~system development	27	2.07E-04
GO:0006796~phosphate metabolic process	17	4.42E-04
GO:0006793~phosphorus metabolic process	17	4.42E-04
GO:0007178~transmembrane receptor protein serine/threonine kinase signaling pathway	6	4.50E-04
GO:0016310~phosphorylation	15	6.81E-04
GO:0001932~regulation of protein amino acid phosphorylation	5	9.47E-04
